# Metabolic engineering of *Bacillus subtilis* for production of *para*‐aminobenzoic acid – unexpected importance of carbon source is an advantage for space application

**DOI:** 10.1111/1751-7915.13403

**Published:** 2019-04-13

**Authors:** Nils J. H. Averesch, Lynn J. Rothschild

**Affiliations:** ^1^ Universities Space Research Association Mountain View CA 94043 USA; ^2^ NASA Ames Research Center Moffett Field CA 94035 USA; ^3^ Present address: Stanford University Stanford CA 94305 USA

## Abstract

High‐strength polymers, such as aramid fibres, are important materials in space technology. To obtain these materials in remote locations, such as Mars, biological production is of interest. The aromatic polymer precursor *para*‐aminobenzoic acid (pABA) can be derived from the shikimate pathway through metabolic engineering of *Bacillus subtilis*, an organism suited for space synthetic biology. Our engineering strategy included repair of the defective indole‐3‐glycerol phosphate synthase (*trpC*), knockout of one chorismate mutase isozyme (*aroH*) and overexpression of the aminodeoxychorismate synthase (*pabAB*) and aminodeoxychorismate lyase (*pabC*) from the bacteria *Corynebacterium callunae* and *Xenorhabdus bovienii* respectively. Further, a fusion‐protein enzyme (*pabABC*) was created for channelling of the carbon flux. Using adaptive evolution, mutants of the production strain, able to metabolize xylose, were created, to explore and compare pABA production capacity from different carbon sources. Rather than the efficiency of the substrate or performance of the biochemical pathway, the product toxicity, which was strongly dependent on the pH, appeared to be the overall limiting factor. The highest titre achieved in shake flasks was 3.22 g l^−1^ with a carbon yield of 12.4% [C‐mol/C‐mol] from an amino sugar. This promises suitability of the system for *in situ* resource utilization (ISRU) in space biotechnology, where feedstocks that can be derived from cyanobacterial cell lysate play a role.

## Introduction and background

Plastics and polymers are not only omnipresent in our everyday life but are potentially of even greater importance in space technology. Biaxially oriented polyethylene terephthalate (BoPET, trade name Mylar^®^) is valued for its high tensile strength, chemical and dimensional stability, barrier properties and electrical insulation; layers of metallized BoPET are, for example, used in high‐altitude balloons as well as in spacesuits for thermal insulation and radiation resistance. Aramids, like the fabric and sheet material Kevlar^®^, feature similarly outstanding properties, including high tenacity and strength modulus, low flex fatigue, as well as excellent chemical stability and thermal stability and also radiation resistance. Therefore, they are ideal for a range of specialty applications, including ballistic protection. That these materials are especially suited for construction of environmental suits and habitations in space technology, shows their utilization in inflatable spacecrafts like the ones of Bigelow Aerospace^®^ (NASA, 2017).

The feedstocks of aromatic polymers are commonly fossil fuel derived, which is neither sustainable in the long run on Earth, nor available in space or at destinations such as Earth's moon or Mars. Metabolic Engineering may provide the technology to solve this problem, by enabling production of bioreplacement precursors through *in situ* resource utilization (ISRU). ISRU aims at utilizing synthetic biology to replenish commodities on deep‐space exploration missions (Rothschild, 2016).

Microbial metabolic pathways give rise to many compounds that can potentially substitute currently petroleum‐based chemicals with bio‐derived ones or replace them with bio‐based alternatives. This includes a multitude of aromatic and aromatic‐derived compounds (Averesch and Krömer, 2018). The shikimate pathway intermediate *para*‐aminobenzoic acid (pABA) is one of these aromatics with versatile applicability – it is being used as cross‐linking agent for resins and dyes, precursor in the pharmaceutical industry and as a therapeutic itself (e.g. as the drug POTABA^®^). pABA can also be converted to terephthalic acid (Farlow and Krömer, 2016), as feedstock for production of PET/Mylar^®^. It may also be possible to convert pABA to *para*‐phenylenediamine (e.g. via Kochi‐ or Hunsdiecker reaction followed by nucleophilic substitution), which is (besides terephthalic acid) the second monomer of the aramid‐fibre Kevlar^®^. Further, pABA can also be polymerized with itself (Morgan, 1977), potentially yielding a *para*‐aramid with a molecular structure analogous to Kevlar^®^.

The feasibility of producing pABA microbiologically to be used as an industrial precursor was first shown using the yeast *Saccharomyces cerevisiae* (Krömer *et al*., 2013), where a titre of 0.03 g l^−1^ (0.22 mM) was reached using glucose as the sole carbon source. In a dedicated follow‐up study, the titre could be increased to 0.22 g l^−1^ (1.57 mM) from glycerol/ethanol (Averesch *et al*., 2016). Also, bacteria have been utilized for the production of pABA. In *Escherichia coli,* a concentration of 4.8 g l^−1^ (35 mM) was reached from glucose (Koma *et al*., 2014), while the highest production to date was accomplished with *Corynebacterium glutamicum*, reaching 43.06 g l^−1^ (314 mM) from glucose (Kubota *et al*., 2016).

To leverage this technology in space and ultimately enable the synthesis of aramid fibres, it would be highly desirable to produce pABA in *Bacillus subtilis*, the organism most suited to space synthetic biology. *Bacillus subtilis* forms endospores (Nicholson *et al*., 2000; Horneck *et al*., 2010), which are extremely resistant to several environmental parameters such as drought, salinity, pH and solvents and remain viable for decades; as long as protected from UV radiation, they even endure the vacuum of space (Horneck, 1993). This allows for convenient long‐term storage of a microbial cell factory while in transit and revitalization for ISRU at destination. Further, *B. subtilis* is able to utilize a large variety of carbon sources, including but not limited to C6 sugar and C5 sugar, organic acids and methanol, while it does not require additional complex supplements (Donnellan *et al*., 1964). Acceptance of a large substrate spectrum is of particular importance, as concepts for ISRU plan for cyanobacteria to form the initial biomass (and thus the carbon source available for most heterotrophic biology) to support autonomous operation of an outpost on Mars (Verseux *et al*., 2015).

The present study provides a holistic picture of how a biological platform technology supporting ISRU for production of pABA may be established.

## Results and discussion

### Toxicity of pABA to *Bacillus subtilis*

**Figure 1:**
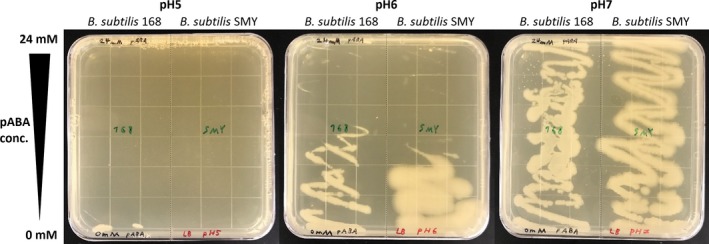
Toxicity of pABA to the *Bacillus subtilis* strains 168 and SMY at different pHs. pABA concentration in the gradient agar plates ranged from 0 mM (bottom‐end of figure) to 24 mM (top‐end of figure), and tested pHs were (from left to right) 5, 6 and 7.

Aminobenzoates have a relatively high toxicity for microorganisms, as compared to other aromatics (Krömer *et al*., 2013). Because toxicity is one of the most restrictive concerns for the biotechnological production of a chemical compound, the toxic effect of pABA on *Bacillus subtilis* 168 and SMY was studied first. Since pABA is an amino acid with different pseudoisomers, growth at different pH levels was assessed, to evaluate the impact of charge states. As shown in Fig. 1, toxicity was high at a pH below 6, while no effect was observed at pH 7 within the tested concentration range of pABA (up to 24 mM). While strain SMY grows in general slightly better (higher density on the plates, higher growth rate in liquid medium – cf., Appendix S2) than 168, the former was slightly more affected by higher concentrations of pABA. Judging from the gradient agar plates, the inhibitory concentration of pABA at pH 6 is slightly above 12 mM for strain 168 and slightly below 12 mM for strain SMY.

The increased toxicity of pABA at lower pH is most likely due to the carboxyl group becoming protonated (pK_a_ = 4.8), allowing diffusion of the uncharged molecule into/through the membrane and (back) into the cell, causing interference. *Corynebacterium glutamicum* was previously shown to be more resistant to pABA than *Saccharomyces cerevisiae*,* Escherichia coli* or *Pseudomonas putida* (Kubota *et al*., 2016), which could be due to its superior amino acid export mechanisms, detoxifying the cell. The reported toxicity limits were around 200–400 mM, which is an order of magnitude higher than the toxicity observed in the present study with *Bacillus subtilis* – however, in the respective previous study, no pH was specified, so that these data are with limited value for comparison. Further, the medium used in the respective previous toxicity study contained glucose, which would have led to formation of the N‐glycosyl of pABA (as described in the same study). The glycosylated form is likely to be much less toxic than pABA itself, analogous to how vanillin toxicity is alleviated by formation of a β‐d‐glucoside in the final step of its biotechnological production (Hansen *et al*., 2009). Glycosylation could thus even be an approach to circumvent the toxicity issue, when producing aromatics like aminobenzoates, besides solvent extraction and mutagenesis/screening of resistant strains (Lee and Wendisch, 2017).

### Choice of strain and genetic construction strategy for production of pABA

Different *B. subtilis* strains were engineered to compare the influence of auxotrophies on growth and pABA production. The defective *trpC*_2_ of the common laboratory strain 168 (Albertini and Galizzi, 1999) was repaired to obtain a prototrophic mutant. In addition, SMY was chosen as a prototrophic derivative for comparison to 168 *trpC* ^+^. SMY is a 168‐W23 hybrid and has common ancestry with PY79 while carrying only one genetic ‘W23 island’ around the *trpC* locus (Zeigler *et al*., 2008).

**Figure 2:**
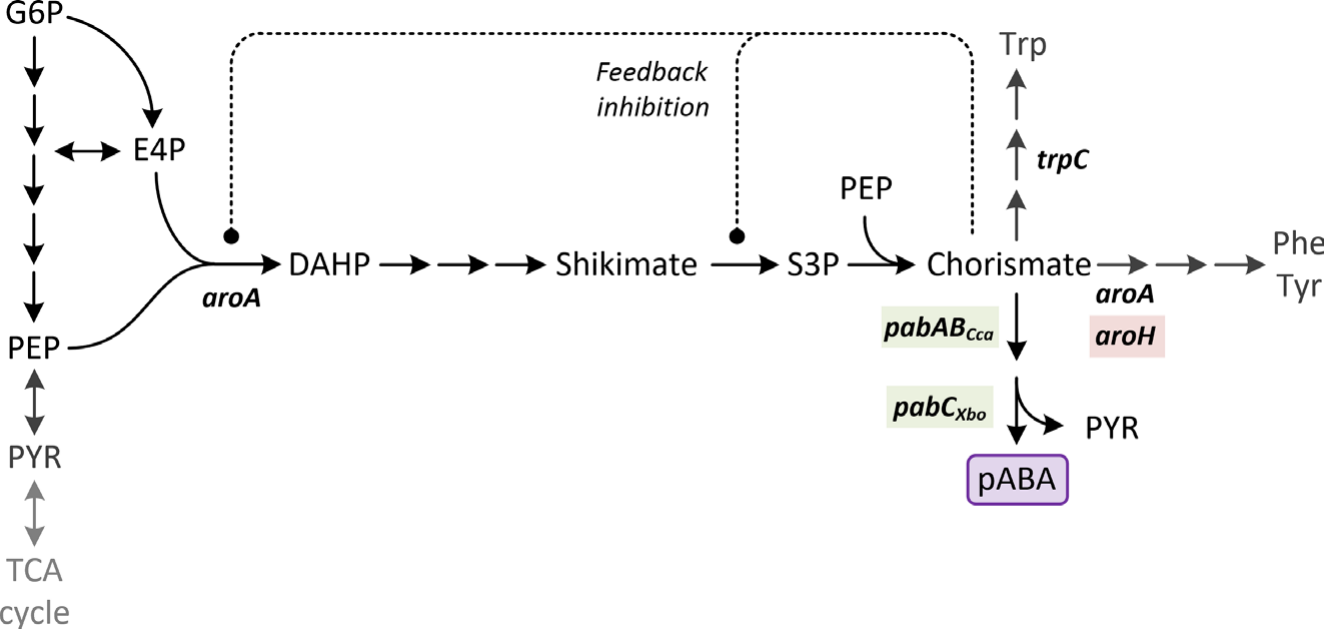
Simplified shikimate pathway and its integration into wider central metabolism, including modifications for pABA production. Knockout targets are highlighted in red, heterological genes that were overexpressed in green. The significant intermediates are glucose 6‐phosphate (G6P), erythrose 4‐phosphate (E4P), phosphoenolpyruvate (PEP), pyruvate (PYR), 3‐deoxy‐d‐arabino‐heptulosonate‐7‐phosphate (DAHP), shikimate, shikimate‐3‐phosphate (S3P), chorismate, phenylalanine (PHE), tyrosine (TYR), tryptophan (TRP) and *para*‐aminobenzoic acid (pABA). Important genes and the respective enzymes involved are *aroA*: 3‐deoxy‐d‐arabino‐heptulosonate‐7‐phosphate (DAHP) synthase/chorismate mutase, *aroH*: chorismate mutase, *trpC*: indole‐3‐glycerol phosphate synthase, *pabAB*: aminodeoxychorismate synthase, *pabC*: aminodeoxychorismate lyase.

The entry step of the shikimate pathway, catalysed by the DAHP synthase, is a popular target in metabolic engineering; in *B. subtilis,* the respective enzyme has also chorismate mutase functionality and is inhibited by chorismate (Wu *et al*., 2005). Here, the partial knockout of the chorismate mutase function by deletion of the *aroH* isozyme was performed to divert flux towards the target product. For formation of pABA from chorismate, the aminodeoxychorismate synthase (*pabAB*) from *Corynebacterium callunae* and the aminodeoxychorismate lyase (*pabC*) from *Xenorhabdus bovienii* have been reported to be most efficient (Kubota *et al*., 2016). For construction of a pABA overproducing strain, two versions of a synthetic operon were designed, a bicistronic design, where *pabAB* and *pabC* were expressed separately and a fusion‐protein design expressing both genes from a single ORF ‘*pabABC’*, spaced by a glycine–serine linker in order to explore the possibility for intermediate channelling (Castellana *et al*., 2014; Sweetlove and Fernie, 2018). The shikimate pathway of *Bacillus subtilis* including the genetic modifications for production of pABA is shown in Fig. 2.

**Figure 3:**
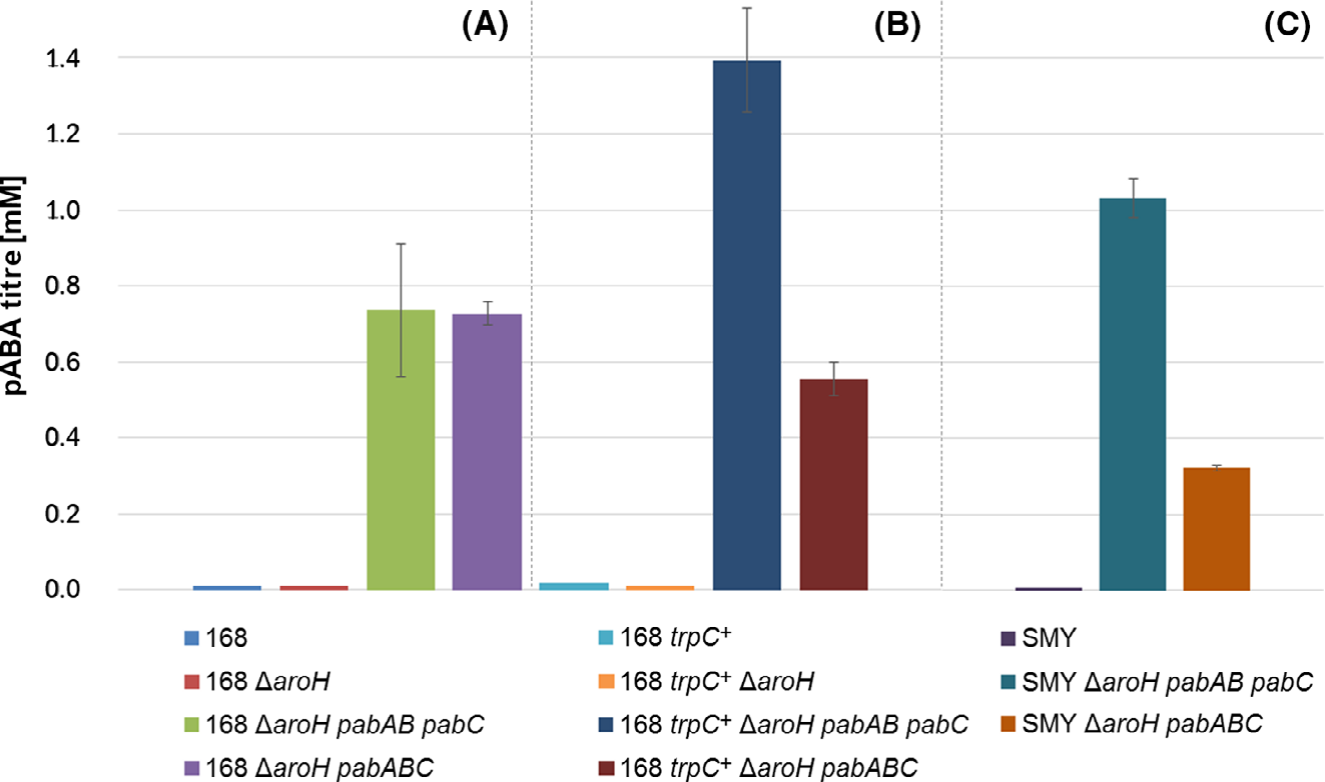
pABA production by differently engineered *B. subtilis* strains. Production from 5 g l^−1^ glucose (≙ 0.1665 C‐mol l^−1^) M9 minimal medium in shake‐flask experiments (end‐point samples shown, taken 12 h after inoculation). Base strains: *B. subtilis* 168 (A) auxotrophic *trpC*_2_, (B) prototrophic *trpC* ^+^ and (C) *B. subtilis* SMY (prototrophic).

The highest titres were achieved with the prototrophic strains (cf., Fig. 3), while pABA production was only detectable in strains carrying the *pab* genes (knockout of *aroH* was not sufficient for accumulation of pABA). In this context, it should be noted that the knockout of *aroH* did not lead to auxotrophy for aromatic amino acids. pABA production from strains carrying the fusion‐protein enzyme could be confirmed but was reduced by about 60% compared to strains expressing the enzymes separately.

For growth of the auxotrophic strains, tryptophan had to be supplemented, which may explain the lower production, as this could lead to feedback inhibition of the shikimate pathway. In case of the strains expressing the *pab* genes, feedback inhibition by chorismate may not be a problem, as substantial flux is drained towards pABA.

Two explanations are possible for the lower pABA production with the fusion‐protein enzyme: (i) loss of activity due to conformational changes/steric hindrance of the active site(s) of the enzyme(s) or (ii) reduced expression of the fusion protein. In search of an answer, the enzymes were tagged (tetracysteine, cf. Appendix S1) to quantify *in vivo* expression levels. pABA production titres were reconfirmed to rule out the possibility of a tag‐inflicted effect on production (data not shown).

**Figure 4:**
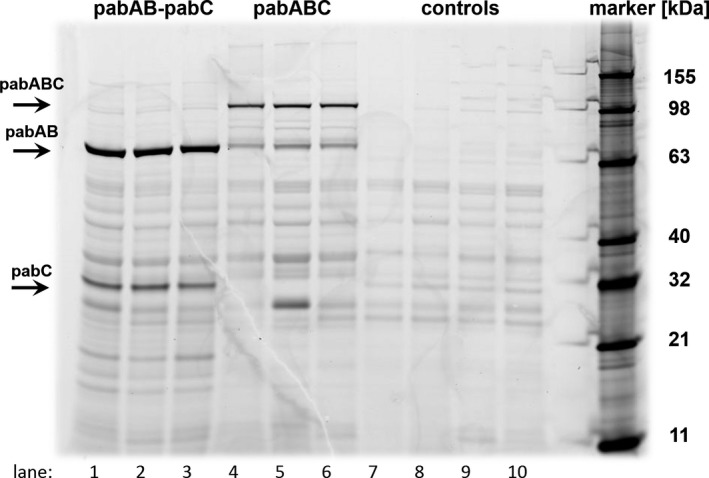
Fluorescence‐based detection of tagged pab enzymes in crude protein extract. Samples were taken from exponential growth phase. Lane 1–3: 168 *trpC* ^+^ Δ*aroH pabAB* ^t^ *pabC* ^t^ (three biological replicates). Lane 4–6: 168 *trpC* ^+^ Δ*aroH pabAB* ^t^ *C* (three biological replicates). Lane 7–10: controls (background strains 168 *trpC* ^+^ and 168 *trpC* ^+^ Δ*aroH*, two biological replicates each).

In the strain expressing the enzymes separately, the amounts of aminodeoxychorismate synthase and aminodeoxychorismate lyase may vary. In contrast, the strain expressing the fusion‐protein enzyme (theoretically) always has the same amounts of both catalysts. This can be used to estimate the relative activity of the catalysts in dependence of their relative abundance. As shown in Fig. 4, the fusion‐protein enzyme *pabABC* is expressed at significantly lower levels than *pabAB*.

The observation that *pabC* is being expressed the lowest (cf., Appendix S4) suggests that this is not the limiting step: the strains with less aminodeoxychorismate lyase function (i.e. the ones expressing the single enzymes) still produce the highest amount of pABA. Therefore, the relative amounts of *pabAB* and *pabABC* may be directly correlated with the production of pABA to evaluate the relative activity of *pabAB* and *pabABC* in respect to the aminodeoxychorismate synthase function. Separately expressed *pabAB* is also detectable in the fusion‐protein strain, likely due to the GC‐rich and repetitive linker–marker sequence joining the two enzymes, which impose difficulties during transcription and/or translation of the protein. Therefore, two relative activities for the aminodeoxychorismate synthase function in the strain carrying the fusion‐protein enzyme were determined (cf., Appendix S3). In average, *pabABC* is 1.10 ± 0.02‐fold as active as *pabAB* when neglecting the *pabAB* fragment and 0.75 ± 0.07‐fold as active as *pabAB* when assuming that the *pabAB* fragment contributes to the total catalytic activity. This means that in the best‐case scenario the fusion protein has slightly higher (≈ 10%) activity, which may be attributable to successful intermediate channelling, while in the worst‐case scenario a loss (≈ 25%) of activity may have occurred, indicating that further optimizations are required. As the fusion protein has a fixed ratio of the two catalytic functions (i.e. one each), the catalytic rate of both needs to be similar in order to avoid a bottleneck and achieve proper channelling. Since *pabAB* appears to be the limiting step, its activity would have to be increased in order to match the flux capacity of *pabC* and create a superior enzyme. As long as the activity of the two catalytic functions differs, individual modulation of expression levels of the single enzymes may present a more promising approach.

### Impact of different carbon sources on production of pABA

*Bacillus subtilis* readily utilizes various carbon sources, including amino sugars like glucosamine and acetyl‐glucosamine (Gaugué *et al*., 2013), but the laboratory strains 168 and SMY cannot utilize xylose. Nevertheless, adaptive laboratory evolution allows strains that grow on xylose to be obtained (Schmiedel and Hillen, 1996). xyl^+^ mutants of the production strains (cf., experimental procedures) were screened in liquid cultures (at least eight per strain), revealing consistent growth (similar to the background strains on glucose), as well as pABA production.

**Figure 5:**
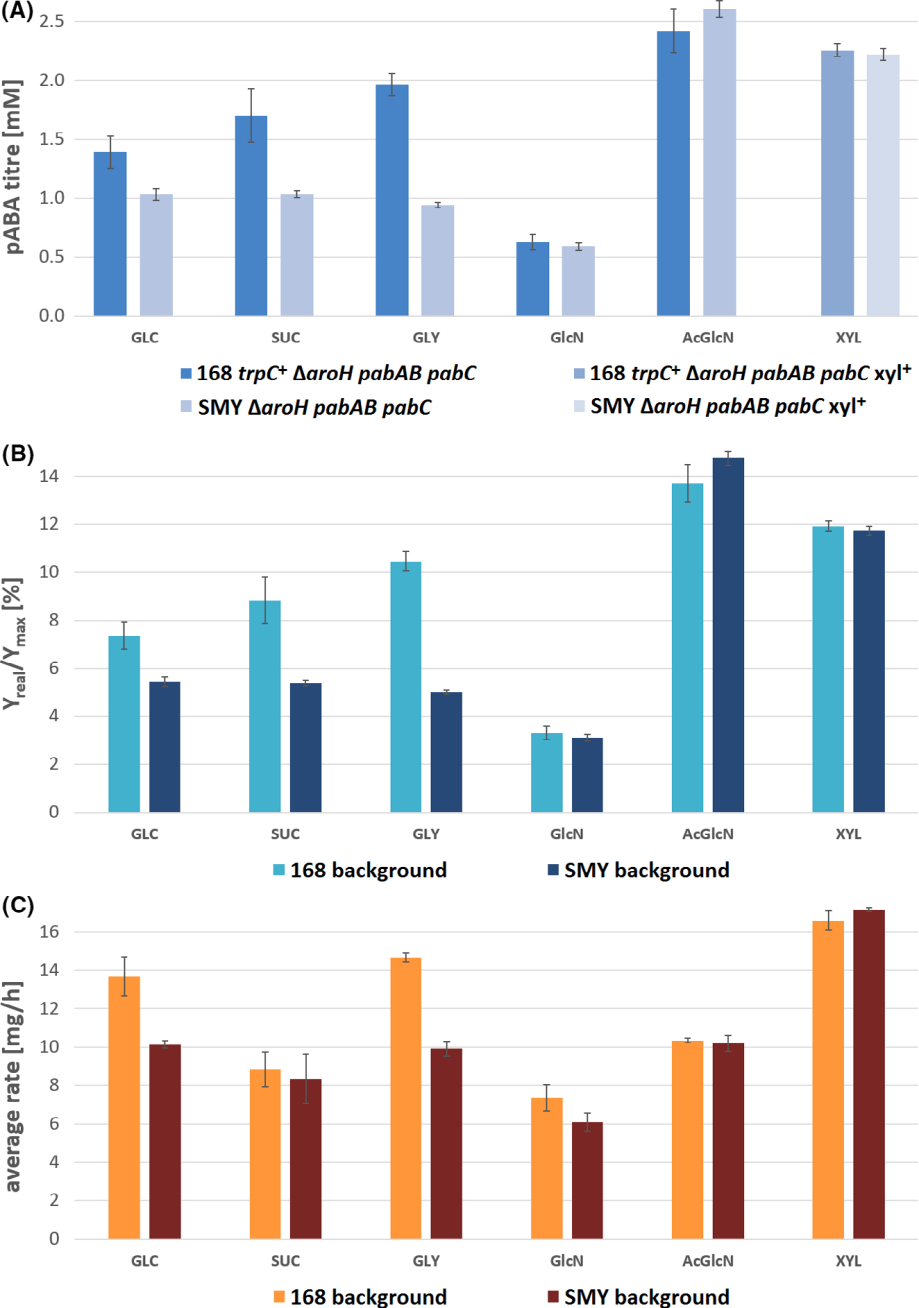
(A–C) pABA production on different carbon sources – titres, yields and rates. Strains 168 *trpC* ^+^ Δ*aroH pabAB pabC* and SMY Δ*aroH pabAB pabC* and their xyl^+^ derivatives were compared on glucose (GLC), sucrose (SUCR), glycerol (GLY), glucosamine (GlcN), acetyl‐glucosamine (AcGlcN) and xylose (XYL) using the equivalent of 5 g l^−1^ glucose (0.1665 C‐mol l^−1^). (A) Shows the maximum achieved titres. (B) The carbon yields. (C) The productivity averaged over the course of the cultivation. Yields are given as fraction of theoretical maximum: *Y*_real_ = carbon yield obtained in shake‐flask experiments with engineered strains (calculated based on supplied carbon source and final product titre), *Y*_max_ = theoretical maximum product yields determined by metabolic modelling (cf., experimental procedures).

Production of pABA by the highest producing strains was compared on different carbon sources, in order to evaluate alternative feedstocks. In particular, sucrose, glycerol, xylose and glucosamine/acetyl‐glucosamine as substrates derivable from sugar cane, biodiesel production, lignocellulose and bacterial lysate were tested. As shown in Fig. 5, the highest titres and yields were achieved on acetyl‐glucosamine and xylose. In particular, the carbon yield on acetyl‐glucosamine was notably high, given the rather low theoretical maximum (cf., Appendix S5). Productivity, however, was low on the amino sugars, while highest on xylose (maximum rate of ≈ 50 mg h^−1^). In most cases, the 168‐based strain performed slightly better than the SMY‐derived strain, except in case of acetyl‐glucosamine as carbon source.

To evaluate the potential of the developed system and determine its limitations, pABA production was examined on increased carbon source, using the two best strains (168 *trpC* ^+^ Δ*aroH pabAB pabC* and SMY Δ*aroH pabAB pabC*) and the three best feedstocks (xylose, glycerol, acetyl‐glucosamine). As shown in Table 1, production on acetyl‐glucosamine is almost 10‐fold higher than on any other carbon source; a maximum titre of 3.22 ± 0.24 g l^−1^ was achieved, with a maximum carbon yield of 12.4% [C‐mol C‐mol^−1^], which is 16.6% of the theoretical maximum. Notably, the SMY‐based strain performed similarly to the 168‐derived strain.

**Table 1:** Comparison of pABA production capacity by the best strains on the best carbon sources at fourfold concentration. Strains 168 *trpC* ^+^ Δ*aroH pabAB pabC* & SMY Δ*aroH pabAB pabC* and their xyl^+^ derivatives were compared on xylose, glycerol and acetyl‐glucosamine using the equivalent of 20 g l^−1^ glucose (0.667 C‐mol l^−1^)

	Xylose (mM)	Glycerol (mM)	Acetyl‐glucosamine (mM)
168 *trpC* ^+^ Δ*aroH pabAB pabC*	2.2 ± 1.1	3.1 ± 1.3	23.5 ± 1.8
SMY Δ*aroH pabAB pabC*	1.2 ± 1.1	1.2 ± 0.3	23.3 ± 0.7

As there is only 1 g l^−1^ NH_4_Cl in M9 medium (equivalent to 18.7 mM ammonium), nitrogen may become limiting when high concentrations of feedstocks that contain only carbon are used for nitrogenous pABA production. Thus, at first sight, it may seem that on acetyl‐glucosamine the additional nitrogen (83.18 mM at 18.4 g l^−1^) allows the approx. 10‐fold higher titre. That this cannot be the reason shows the example of production on glucosamine, where even more nitrogen is available (additional 100.46 mM at 18 g l^−1^), but the final pABA titre is the lowest. To validate this hypothesis, an experiment on sucrose with increased availability of carbon (0.667 C‐mol) and nitrogen (100 mM from NH_4_Cl) was carried out. As well growth as also production of pABA appeared to be slightly increased (higher final biomass, cf. Appendix S2, 3.2/2.3 mM final pABA titre for 168 and SMY, respectively), but not in the same range as from acetyl‐glucosamine. The explanation for the significantly higher production of ≈ 24 mM pABA (which is somewhat surprising, given the toxicity‐limit of 12 mM at pH 6) on medium with acetyl‐glucosamine is a different phenomenon and can be explained when studying the trend of the pH throughout the cultivations: while the pH drops below 6 on all but one carbon source, it rises to above 7 during growth on acetyl‐glucosamine (cf., Appendix S2). Thus, pABA is significantly less toxic in cultivations on acetyl‐glucosamine, allowing higher production. This may also explain why production by the SMY‐derived strains is always lower than with 168‐based strains, unless when grown on acetyl‐glucosamine: because SMY is slightly more sensitive to pABA (cf., Fig. 1), it is inhibited more at lower pH, while this effect is less pronounced at higher pH.

While on non‐acetylated carbon sources, the pH drops, due to production of organic acids as metabolic end‐products, the pH rises during growth on acetyl‐glucosamine, as the acetylated carbon source is consumed, and acid is eliminated from the medium. The direct coupling of a preferred with a non‐preferred carbon source may also explain the much slower growth, as both need to be metabolized at the same rate (Gaugué *et al*., 2013).

## Conclusion

*Bacillus subtilis* was engineered to produce pABA. Production was demonstrated from various carbon sources, at the highest resulting in a titre of 3.22 g l^−1^ with a yield of 12.4% [C‐mol C‐mol^−1^] despite the observed high toxicity of pABA. This could be accomplished with acetyl‐glucosamine as a substrate, which led to a rise of the pH over the course of the cultivation, significantly reducing end‐product toxicity. The pH effect of better production on acetyl‐glucosamine is an advantage for ISRU and an opportunity for earth‐based production: for application in remote places, no pH control is required, eliminating requirements for additional equipment and resources (in particular neutralization). pH control imposes not only a limitation for in‐space production, but can be a significant cost factor in an industrial‐scale process, as the example of lactic acid production shows (Juturu and Wu, 2016; Komesu *et al*., 2017).

Acidic amino sugars are the monomers of the polysaccharide part of peptidoglycan (which constitutes the bacterial cell wall, also known as murein sacculus), so that these amino sugars can be also found in cyanobacterial cell lysate. This makes the here developed microbial cell factory promising for ISRU in space biotechnology. Further, amino sugars are abundant in nature, specifically found in the form of the polysaccharides chitosan, chitin and murein. As amino sugars are thus not plant‐derived, these feedstocks do also not directly compete with food sources and may therefore potentially be a sustainable feedstock (supplement) in earth‐based biotechnology.

## Experimental procedures

### Strains, media and cultivation

*Bacillus subtilis* strains were obtained from the Bacillus Genetic Stock Center (BGSC; http://www.bgsc.org/). Growth conditions were 37°C on solid or liquid LB or M9 medium. In case of liquid cultures, baffled shake flasks were filled with no more than 10% culture and incubated with shaking at 220 RPM in an Innova 4230 refrigerated benchtop incubator (New Brunswick Scientific, Enfield, CT, USA) to keep dissolved oxygen above 30%. Growth was characterized by measuring the optical density at 650 nm (OD_650_) with a SpectraMax Plus 384 (Molecular Devices, San Jose, CA, USA). A comprehensive list of the strains used and constructed in this study is shown in Table 2.

**Table 2:** *Bacillus subtilis* strains used and engineered in this study

Strain/plasmid	Description/genotype	Origin/BGSC catalogue number
168	*B. subtilis* subsp. *subtilis*, common laboratory strain, auxotrophic (*trpC*_2_)	1A1
NCIB 3610^T^	Putative parent strain of *B. subtilis* 168, prototrophic	3A1
SMY	Prototrophic W23–168 hybrid strain, one genomic island of W23 around the *trpC* locus, shares ancestry with PY79	1A775
168 *trpC* ^+^	168 with *trpC* of NCIB 3610^T^	This study/1A900
168 Δ*aroH*	168 with erythromycin resistance cassette, integrated at the *aroH* locus (*erm*HI::*aroH*)	BKE22690
168 Δ*aroH trpC* ^+^	BKE22690 with *trpC* of NCIB 3610^T^	This study
168 Δ*aroH pabAB pabC*	168 *pabAB*_Cca_‐*pabC*_Xbo_‐*erm*HI::*aroH*	This study
168 *trpC* ^+^ Δ*aroH pabAB pabC*	168 *trpC*_NCIB3610_ *pabAB*_Cca_‐*pabC*_Xbo_‐*erm*HI::*aroH*	This study
168 *trpC* ^+^ Δ*aroH pabAB pabC* xyl^+^	168 *trpC*_NCIB3610_ *pabAB*_Cca_‐*pabC*_Xbo_‐*erm*HI::*aroH*, xylose adapted	This study
168 *trpC* ^+^ Δ*aroH pabABC*	168 *trpC*_NCIB3610_ *pabAB*_Cca_ *C*_Xbo_‐*erm*HI::*aroH*	This study
168 *trpC* ^+^ Δ*aroH pabAB pabC*	168 *trpC*_NCIB3610_ *pabAB*_Cca_‐*pabC*_Xbo_‐*erm*HI::*aroH*	This study
168 *trpC* ^+^ Δ*aroH pabAB* ^t^ *pabC* ^t^	168 *trpC*_NCIB3610_ *pabAB*_Cca_^TC^‐*pabC*_Xbo_^TC^‐*erm*HI::*aroH*	This study
168 *trpC* ^+^ Δ*aroH pabABC* ^t^	168 *trpC*_NCIB3610_ *pabAB*_Cca_^TC^ *pabC*_Xbo_‐*erm*HI::*aroH*	This study
SMY Δ*aroH pabAB pabC*	SMY *pabAB*_Cca_‐*pabC*_Xbo_‐*erm*HI::*aroH*	This study
SMY Δ*aroH pabAB pabC* xyl^+^	SMY *pabAB*_Cca_‐*pabC*_Xbo_‐*erm*HI::*aroH*, xylose adapted	This study

LB Broth (Lennox) was made up from pre‐buffered capsules (Fisher BioReagents™, Pittsburgh, PA, USA). When applicable, erythromycin (Sigma, St. Louis, MO, USA), lincomycin (Sigma) or carbenicillin (Sigma) was used in a concentration of 2, 200 and 100 μg ml^−1^ respectively.

One litre of minimal medium (M9) contained 200 ml of 5× M9 minimal salts stock solution (Sigma), 10 ml of 100× trace element stock solution, 25/200 ml of carbon source stock solution, 763/588 ml water, 1 ml of 1000 × 100 mM CaCl_2_ stock solution and 1 ml of 100 × 1 M MgSO_4_ stock solution. For the auxotrophic strains, tryptophan (Sigma) was added to a final concentration of 50 mg l^−1^. The M9 minimal salt stock solution contained 33.9 g l^−1^ Na_2_HPO_4_, 15 g l^−1^ KH_2_PO_4_, 5 g l^−1^ NH_4_Cl and 2.5 g l^−1^ NaCl. The trace element stock solution contained 100 mg l^−1^ MnCl_2_·4H_2_O, 450 mg l^−1^ ZnSO_4_·7H_2_O, 30 mg l^−1^ CuSO_4_·5H_2_O, 30 mg l^−1^ CoCl_2_ ·6H_2_O, 30 mg l^−1^ Na_2_MoO_4_·2H_2_O, 1400 mg l^−1^ FeSO_4_·7H_2_O, 100 mg l^−1^ H_3_BO_3_, 10 ng l^−1^ KI and 1.5 g l^−1^ of EDTA. Carbon sources were used in a concentration of 0.1665 C‐mol or 1.332 C‐mol, so that stocks were 200 g l^−1^ glucose (Sigma), 190 g l^−1^ sucrose (Sigma), 200 g l^−1^ xylose (Sigma), 204.4 g l^−1^ glycerol (Sigma), 184 g l^−1^ N‐acetyl‐d‐glucosamine (BULKSUPPLEMENTS.COM) and 180 g l^−1^ glucosamine HCL (BULKSUPPLEMENTS.COM). Solid medium contained 16 g agar (Sigma).

Gradient agar plates (LB) for pABA‐toxicity experiments were prepared by pouring two layers of agar. The first layer contained medium with the highest pABA concentration (24 mM), poured into a slanted square Petri dish so that the medium filled approximately 2/3 of the plate on the lowered side, while barely covering at the opposing (elevated) side. The agar was allowed to solidify, and the plates were positioned at level to be filled with another layer of medium without pABA, which covered 100% at one side of the plate and 0% at the opposing side, to obtain an even level of total medium in the dish. Thus, through vertical diffusion, plates with a pABA concentration ranging from 0 mM on one side to 24 mM on the opposing side were obtained. The procedure was the same for all tested pHs. The pH of the buffered medium was adjusted before autoclaving, while pH‐adjusted pABA stock was only added after autoclaving, to avoid degradation. Gradient plates were used within 24 h, in order to avoid significant horizontal diffusion of pABA.

In *B. subtilis*, genetic competence develops post‐exponentially as a global response to the onset of stationary phase in glucose‐minimal salt‐based media. Still, only a minority of the cells become competent (Dubnau, 1991). Two different liquid media were used in the transformation of *B. subtilis*. Medium A contained 2 g l^−1^ yeast extract, 0.25 g l^−1^ casamino acids, 2 g l^−1^ (NH_4_)_2_SO_4_, 14 g l^−1^ K_2_HPO_4_, 6 g l^−1^ KH_2_PO_4_, 0.7 g l^−1^ Na‐citrate, 0.1 g l^−1^ MgSO_4_, 5 g l^−1^ glucose. Medium B contained: 1 g l^−1^ yeast extract, 0.1 g l^−1^ casamino acids, 2 g l^−1^ (NH_4_)_2_SO_4_, 14 g l^−1^ K_2_HPO_4_, 6 g l^−1^ KH_2_PO_4_, 0.7 g l^−1^ Na‐citrate, 0.4 g l^−1^ MgSO_4_, 0.5 g l^−1^ CaCl_2_ and 5 g l^−1^ glucose. The transformation protocol was based on the established method (Yasbin *et al*., 1975); specifically, 25 ml of medium A was inoculated to an OD_650_ of ≈ 0.2 from a spore plate and incubated at 37°C with shaking (baffled shake flask), while monitoring growth. After cessation of exponential growth and 90 more minutes, 0.5 ml of the culture was transferred to 4.5 ml of pre‐warmed medium B and incubated at 37°C with shaking (baffled shake flask). After another 90 min at 37°C, aliquots of 0.5 ml of the culture were immediately used for transformation (15‐ml screw‐cap tubes): at the minimum 1 μg (10 μg for strain SMY) of highly concentrated DNA was added per 0.5 ml culture and the mixture was incubated at 37°C. After 60–90 min, the tubes were centrifuged at 4000 *g* for 2 min to separate the supernatant, which was subsequently removed. The cell pellet was resuspended in 100–200 μl of sterile H_2_O, and the suspension was immediately plated on selective agar.

Erythromycin plates were replica‐plated on lincomycin plates to select for resistance to the full spectrum of MLS antibiotics, rather than a ribosomal mutant. Colonies were picked and screened on successful integration of the construct by means of colony PCR, using primers that bind inside and outside of the target region and construct, to allow for positive and negative controls (see Appendix S1 for primer sequences and binding sites). Candidate colonies that passed genetic screening were selected (minimum of three) for testing on pABA production in minimal medium.

### Genetic engineering

The defective *trpC*_2_ of strain 168 was repaired by amplifying the *trpC* of strain NCIB 3610^T^ (purified gDNA was used as template, prepared with the phenol–chloroform method (Sambrook and Russell, 2006)) and transforming strain 168 with the PCR product. Prototrophic mutants were selected on chemically defined medium plates, omitting tryptophan to select for prototrophic mutants.

Sequences of *pabAB* from *Corynebacterium callunae* and *pabC* from *Xenorhabdus bovienii* (Kubota *et al*., 2016) were codon‐optimized for expression in *B. subtilis*. Expression operating units (promoter, transcription start site, ribosomal binding‐site, terminator, spacer sequences) were derived from literature (Guiziou *et al*., 2016). The genetic construct harboured an *erm*HI cassette (Koo *et al*., 2017) for antibiotic‐based selection and was flanked with 700‐bp regions of the target locus for homologous recombination. The integration site on the genome between bases 2 377 619 and 2 378 012 was chosen to replace *aroH*, the last gene in the respective operon. The genome sequence of *B. subtilis* (Barbe *et al*., 2009) was derived from BsubCyc (Caspi *et al*., 2014, 2016).

The construct was assembled on pUC19 by fusing the flanking regions with two custom synthesized DNA fragments. Specifically, fragment 1 consisted of *pabAB* with regulatory elements while fragment 2 comprised *pabC* with regulatory elements and the *erm*HI (erythromycin resistance) cassette. The construct was designated ‘vector A’ and sequence verified before production of linear DNA by PCR for integration into the *B. subtilis* genome. The vector for expression of the fusion‐protein enzyme ‘vector B’ was constructed by amplifying a linear fragment of vector A via PCR round amplification from the beginning of *pabC* to the end of *pabAB* and recircularization with a custom synthesized linker fragment. A detailed description of the used primers, vectors and genetic constructs with their expression operating units (full sequences of the vectors) is shown in Appendix S1.

Gene fragments (gBlocks) were purchased from Integrated DNA Technologies, oligonucleotides were purchased from Elim Biopharmaceuticals, Inc., Q5 polymerase (NEB) was used in all PCR applications, and Gibson assembly was performed with NEBuilder (NEB). PCR products were purified using the QIAquick^®^ Gel Extraction Kit (Qiagen, Hilden, Germany), and plasmids were extracted using the GeneCatch™ Plus Plasmid Miniprep Kit (Epoch, Missouri City, TX, USA). Sequencing service was provided by Elim Biopharmaceuticals, Inc.

Chemically competent NEB^®^ 5‐alpha (DH5α™ derivative) *E. coli* cells, obtained from New England BioLabs^®^ Inc., were used for all molecular cloning applications.

### Adaptive evolution for xyl^+^ strain construction

When plating xyl^−^ *B. subtilis* 168 and SMY strains on xylose‐containing minimal medium, colonies appear with a frequency of approximately 1 × 10^−6^/cell (Schmiedel and Hillen, 1996). To evaluate production of pABA from xylose, adaptive evolution was performed with the two best pABA producers: 168 *trpC* ^+^ Δ*aroH pabAB pabC* and SMY Δ*aroH pabAB pabC*. Picking and restreaking of colonies revealed xyl^+^ mutants (see Appendix S4 for illustrating pictures), leading to the strains 168 *trpC* ^+^ Δ*aroH pabAB pabC* xyl^+^ and SMY Δ*aroH pabAB pabC* xyl^+^. In particular, three specific mutations have been identified to benefit growth on xylose (Zhang *et al*., 2015).

### Colorimetric quantification of aminobenzoates

pABA concentration in aqueous solutions can be analysed colorimetrically using the *PABAcheck* method; the assay is based on formation of a diazonium salt with a coupling agent (Bingham and Cummings, 1983; Williams and Bingham, 1986; Sharma *et al*., 2014). Originally developed to assess completeness of 24‐h urine collections, the method was found to be also specific as well as sensitive enough for quantification of aminobenzoates from minimal microbial media, such as M9. The assay is sensitive to *para*‐aminobenzoate, *meta*‐aminobenzoate, *ortho*‐aminobenzoate, 4‐amino‐phenylalanine and benzocaine, but not to other amino acids derived from the shikimate pathway, such as tryptophan, phenylalanine or tyrosine. For pABA, the correlation between dye strength (absorption at 540 nm) and concentrations of the compound is linear up to about 40 μM, while from 40 μM to concentrations as high as 120 μM a quadratic dependency results in an excellent fit. The correlations between concentration (for *para*‐aminobenzoate, *meta*‐aminobenzoate, *ortho*‐aminobenzoate, 4‐amino‐phenylalanine and benzocaine) and absorption are shown in Appendix S3.

The protocol for the preparation of samples was shortened and adapted for microcuvettes: pABA standards (serial dilution) of 128, 64, 32, 16, 8, 4, 2, 1 μM were prepared from stock, every time the assay was conducted. To 500 μl of each standard or sample, 50 μl of 5 M HCl was added to drop the pH below 2 (necessary condition for reaction, also converts any N‐glucosyl (Kubota *et al*., 2016) to free aminobenzoate). Subsequently, 50 μl of a 14.5 mM NaNO_2_ (sodium nitrite) solution was added; samples and standards were mixed and incubated at room temperature for 2–20 min. Then, 50 μl of 44 mM H_6_N_2_O_3_S (ammonium sulphamate) solution was added; after mixing, the cuvettes were incubated at room temperature for 2–3 min to stop the reaction. For formation of the azo‐dye, 50 μl of 4 mM N‐(1‐naphthyl)‐ethylenediamine dihydrochloride solution was added; colour was allowed to develop for at least min 20 min at room temperature, before absorbance was measured at 540 nm. In case, absorbance was outside of the confidence range (1–120 μM); the assay was repeated with samples diluted with water.

The assay can potentially also be miniaturized for analysis in microtitre plates. It should be noted that extended incubation (60 min) in the last step increases intensity of the pigment but will eventually lead to precipitation at pABA concentrations greater than 100 μM (after 24 h or sooner). *Ortho*‐aminobenzoate, *meta*‐aminobenzoate, benzocaine and 4‐amino‐phenylalanine, however, form stable azo‐dyes at concentrations of 1 mM, potentially also higher. Further, the N‐(1‐naphthyl)‐ethylenediamine dihydrochloride solution, as well as the aminobenzoate stock solutions (10 mM), slowly degrades at room temperature, which affects dye stability and intensity and thus affects the calibration curve. For these reasons, stocks of these compounds were stored frozen.

### Protein extraction and detection

Culture derived from distinct time‐points of a batch cultivation (exponential‐phase/stationary‐phase) was collected (sample volume [ml] ≈ 10/OD_650_), and cells were pellet by centrifugation (7800 *g* at 4°C for 5 min). The pellet was washed with mQ H_2_O and stored as cell paste at −20°C for later processing. For extraction of proteins, CelLytic™ B (Sigma) was used as per manufacturer's directions (approx. 1 ml per cells from 10 ml culture at an OD of 1), in combination with Protease Inhibitor Cocktail (Sigma‐Aldrich). In addition, the mixture was sonicated for 2 min (on ice) to lyse cells and extract the soluble proteins, using a Misonix Sonicator XL Ultrasonic Processor set to 2‐s pulse with 0.5‐s pause at 20–30% output. Centrifugation (15 000 *g* for 10 min) pelleted the cell debris; the supernatant, which contained the soluble proteins, was separated. Total protein concentration was determined using the BCA Protein Assay Kit (Pierce™, Waltham, MA, USA). Using the Lumio™Green Detection Kit (ThermoFisher, Waltham, MA, USA) as per manufacturer's directions, 10 μg crude protein extract of each sample was prepared for gel electrophoresis. Size separation was performed on a Bolt™ 4–12% Bis‐Tris Plus Gel (ThermoFisher), run at 150 V for approx. 40 min with Bolt™ MES SDS Running Buffer (ThermoFisher). The marker was BenchMark™ Fluorescent Protein Standard (ThermoFisher). A laser‐based scanner (Typhoon TRIO Variable Mode Imager) was used to detect fluorescent‐conjugated proteins at 488 nm excitation with a 526‐nm emission filter. For visualization of all proteins, the gel was restained with SYPRO Orange (ThermoFisher) as per manufacturer's directions and imaged again as before. Analysis and intensity quantification of bands were performed with myImageAnalysis™ Software (ThermoFisher). Expression levels of the aminodeoxychorismate lyase (*pabC*) and aminodeoxychorismate synthase/lyase fusion protein (*pabABC*) were determined relative to the expression of aminodeoxychorismate synthase (*pabAB*). Specifically, band intensities as fold of reference band intensities were determined from triplicates (biological replicates), on different carbon sources (sucrose and acetyl‐glucosamine) and at different stages throughout the cultivations (exponential‐phase and stationary‐phase). The values of the resulting relative quantities and errors are shown in Appendix S4. To determine relative activity of the respective enzymes, the amount of pABA produced per amount of catalyst was calculated, as the quotient of fold‐pABA production and fold enzyme expression. The resulting fold activities can also be found in Appendix S4.

### Metabolic modelling

A base metabolic network describing central carbon metabolism of *B. subtilis* (glycolysis, pentose phosphate pathway, TCA‐cycle, fermentation, anaplerosis, redox/energy metabolism) was derived from literature (Unrean and Nguyen, 2013), reconstructed and amended with the different substrate utilization pathways using BsubCyc (Caspi *et al*., 2014, 2016) as well as the product pathway (Averesch *et al*., 2016). The final networks comprised 80–86 reactions, which included interconversion of metabolites, biomass function and transport across the system boundaries.

The theoretical maximum product yields were computationally determined by means of elementary flux mode analysis (Terzer and Stelling, 2008). This method calculates all feasible steady‐state flux distributions in a metabolic network, that is elementary modes. Specifically, the metabolic networks were parsed into stoichiometric matrices in MATLAB^®^ (MathWorks^®^) (RRID:SCR_001622) using EFMTool (Terzer and Stelling, 2008) (RRID:SCR_016289) and solutions for each network were calculated using the recent implementation FluxModeCalculator (van Klinken and Willems van Dijk, 2015) (RRID:SCR_016290). The results were evaluated as described before (Averesch and Krömer, 2014); briefly, the maximum carbon yields of the different elementary modes were determined by drawing carbon balances around the transport reactions into and out of the network. The yield for each product in each mode is defined by: Yield_product_ = Flux_product_ × Carbon_product_/Flux_substrate_ × Carbon_substrate_, where Flux_product_ and Flux_substrate_ represent the flux rates for products and substrates leaving and entering the balance area, and Carbon_product_ and Carbon_substrate_ are the numbers of carbon atoms in the product and substrate. The networks and calculated carbon yields (converted to per cent) are shown in Appendix S5.

## Conflict of interests

The authors declare that they have no competing interests.

## Authors' contributions

NHJA and LJR jointly conceived the study. NHJA designed and conducted the experiments, analysed and interpreted the data and drafted the manuscript. LJR edited the manuscript. Both authors read and approved the final manuscript.

## Supporting information


**Appendix S1.** In detail description of genetic constructs, primer and DNA sequences.

**Appendix S2.** Cultivation data containing growth‐curves and development of the pH.

**Appendix S3.** Protocol of aminobenzoate‐assay and example standard‐curves.

**Appendix S4.** Additional data of protein quantification and figures of xyl^+^ mutants.

**Appendix S5.** EMA networks and theoretical maximum product carbon‐yields.
