# Supplementary material for: Metabolic engineering of Bacillus subtilis for production of para‐aminobenzoic acid – unexpected importance of carbon source is an advantage for space application
Source: Microb Biotechnol. 2019 Apr 13;12(4):703–14. doi: 10.1111/1751-7915.13403 (PMC6559200; doi:10.1111/1751-7915.13403)
Supplement: Supplementary file 1 — Appendix S1. In detail description of genetic constructs, primer and DNA sequences. [file MBT2-12-703-s001.pdf]

Architecture of vectors for expression of *pabAB* and *pabC*

A genetically insulated synthetic operon was designed to replace the *aroH* locus (393 bp ORF, last gene in the respective operon) on the *B. subtilis* chromosome. In addition to the bicistronic design (vector A), a fusion-protein (vector B) was designed. Vector A was assembled from five fragments (two gene-blocks comprised of ORFs and regulatory elements – gBlock1: *pabAB*, gBlock2: *pabC* + marker, and two fragments of 5’ and 3’ flanking regions for homologous recombination) and sub-cloned into pUC19 for sequence verification and convenient amplification of the construct. Vector B was assembled by inserting a linker into linearized vector A. The vectors encoding tagged pab-proteins were constructed the same way.

***aroH* locus of *B. subtilis* 168 before integration:**

atgAAGACACTGCATGTTCAAACTGCTTCCTCGTCATATCCTGTTTTTATCGGACAAGGTATCAGAAAAAAGGCTTGCGAAC
TATTAACCTCTTTAAACAGGCCTTTAACAAGGATTATGTTTGTCACGGATGAAGAAGTGGACCGCCTGTATGGTGATGAAAT
GCTCCATTTGCTGCAAGAAAAATGGCCTGTGAAAAAAGTGACGGTACCGAGTGGAGAGCAGGCAAAATCCATGGACATGTAC
ACAAAATTGCAGAGTGAAGCGATTCGTTTTCATATGGATCGCTCCTCATGCATCATCGCATTCGGTGGCGGTGTTGTAGGGG
ATCTTGCCGGTTTTGTGGCTGCCACTTTTATGCGCGGCATTGATTTCATCCAAATGCCTACGACGCTCTTGGCGCATGACAG
CGCAGTCGGAGGAAAAGTAGCCGTGAACCATCCGCTTGGAAAAAATCTAATCGGTGCGTTTTATCAGCCGAAAGCCGTGCTC
TATGATACAGATTTCCTGCGTTCTCTGCCTGAAAAAGAGCTTAGGTCCGGAATGGCAGAAGTGATTAAACACGCTTTTATCT
ATGACAGAGCGTTTCTGGAGGAGCTGCTGAATATCCACTCACTGCGTGATATCACTAATGATCAGCTGAATGACATGATTTT
TAAAGGCATTTCAATTAAAGCGTCTGTCGTTCAGCAGGATGAAAAAGAAGAGGGGATAAGAGCTTACTTAAACTTTGGGCAT
ACGCTCGGCCATGCCGTTGAGGCGGAATATGGATATGGGCAGATCACTCACGGTGATGCCGTAGCCCTCGGAATGCAGTTTG
CTTTATATATAAGCGAAAAGACTGTAGGCTGTGAAATGGACAGAAAACGTTTGGTCAGCTGGCTAAAAAGCCTGGGTTATCC
AAGTCAAATCAGAAAAGAGACGGAAACTTCGGTTCTCTTGAACCGTATGATGAATGATAAGAAAACCCGTGGCGGGAAGATT
CAGTTTATTGTGCTCAACGAATTAGGGAAAGTTGCTGATCATACATTTTCCAGAAATGAACTTGAGAGCTGGCTGAACAAAT
GGCGATTGGAGGAGACATCatgaTGATTCGCGGAATTCGCGGAGCAACTACAGTTGAACGGGATACTGAAGAAGAAATTTTA
CAAAAAACTAAACAGCTGTTAGAGAAAATCATAGAAGAAAATCATACAAAACCGGAAGATGTTGTTCAAATGCTTCTGTCGG
CTACACCTGATTTGCACGCTGTTTTCCCGGCAAAAGCTGTTCGCGAGCTTTCAGGATGGCAGTATGTACCGGTAACATGTAT
GCAGGAAATGGACGTAACAGGCGGTCTGAAAAAGTGCATAAGAGTCATGATGACGGTCCAGACAGATGTCCCTCAGGATCAG
ATCAGACATGTATATTTAGAAAAAGTTGTCGTATTGAGGCCCGATTTATCATTGACAAAAAATACTGAATTGtaaTACGATA
AGAACAGCTTAGAAATACACAAGAGTGTGTATAAAGCAATTAGAATGAGTTGAGTTAGAGAATAGGGTAGCAGAGAATGAGT
TTAGTTGAGCTGAGACATTATGTTTATTCTACCCAAAAGAAGTCTTTCTTTTGGGTTTATTTGTTATATAGTATTTTATCCT
CTCATGCCATCTTCTCATTCTCCTTGCCATAAGGAGTGAGAGCAatgAATTTCCAATCAAACATTTCCGCATTTTTAGAGGA
CAGCTTGTCCCACCACACGATACCGATTGTGGAGACCTTCACAGTCGATACACTGACACCCATTCAAATGATAGAGAAGCTT
GACAGGGAGATTACGTATCTTCTTGAAAGCAAGGACGATACATCCACTTGGTCCAGATATTCGTTTATCGGCCTGAATCCAT
TTCTCACAATTAAAGAAGAGCAGGGCCGTTTTTCGGCCGCTGATCAGGACAGCAAATCTCTTTACACAGGAAATGAACTAAA
AGAAGTGCTGAACTGGATGAATACCACATACAAAATCAAAACACCTGAGCTTGGCATTCCTTTTGTCGGCGGAGCTGTCGGG
TACTTAAGCTATGATATGATCCCGCTGATTGAGCCTTCTGTTCCTTCGCATACCAAAGAAACAGACATGGAAAAGTGTATGC
TGTTTGTTTGCCGGACATTAATTGCGTATGATCATGAAACCAAAAACGTCCACTTTATCCAATATGCAAGGCTCACTGGAGA
GGAAACAAAAAACGAAAAAATGGATGTATTCCATCAAAATCATCTGGAGCTTCAAAATCTCATTGAAAAAATGATGGACCAA
AAAAACATAAAAGAGCTGTTTCTTTCTGCTGATTCATACAAGACACCCAGCTTTGAGACAGTATCTTCTAATTATGAAAAAT
CGGCTTTTATGGCTGATGTAGAAAAAATCAAAAGCTATATAAAAGCAGGCGATATCTTCCAGGGTGTTTTATCACAAAAATT
TGAGGTGCCGATAAAAGCAGATGCTTTTGAGTTATACCGAGTGCTTAGGATCGTCAATCCTTCGCCGTATATGTATTATATG
AAACTGCTAGACAGAGAAATAGTCGGCAGCTCTCCGGAACGGTTAATACACGTTCAAGACGGGCACTTAGAAATCCATCCGA
TTGCCGGTACGAGAAAACGCGGTGCAGACAAAGCTGAAGATGAGAGACTGAAGGTTGAGCTCATGAAGGATGAAAAAGAAAA
AGCGGAGCATTACATGCTCGTTGATCTTGCCCGAAACGATATCGGCAGAGTAGCAGAGTATGGTTCTGTTTCTGTGCCGGAG
TTCACAAAAATTGTTTCCTTTTCACATGTCATGCACATTATCTCGGTGGTTACAGGCCGATTGAAAAAAGGGGTTCATCCTG
TCGATGCACTGATGTCTGCTTTCCCGGCGGGGACTTTAACAGGCGCACCCAAAATCCGTGCCATGCAGCTTTTGCAAGAACT
CGAGCCAACACCGAGAGAGACATACGGAGGGTGTATTGCCTACATTGGGTTTGACGGGAATATCGACTCTTGTATTACGATT
CGCACGATGAGTGTAAAGAACGGTGTTGCATCGATACAGGCAGGTGCTGGCATTGTTGCTGATTCTGTTCCGGAAGCCGAAT
ACGAAGAAAGCTGTAATAAAGCCGGTGCGCTGCTGAAAACGATTCATATTGCAGAAGACATGTTTCATAGCAAGGAGGATAA
AGCTGATGAACAGATTTCTACAATTGTGCGTtga

***aroH* locus** of *B. subtilis* 168 after integration of

Vector A (bicistronic design): aroB_FR-5'OH-T_{B0014}-spacer$_1$-P_{ymdA}-TSS-RBS$_0$-*pabAB*$_{Cca}$-spacer$_2$-RBS$_1$-*pabC*$_{Xbo}$-spacer$_3$-T_{B0015}-lox71-**erm**^{R}-lox66-3'OH-trpE_FR

atgAAGACACTGCATGTTCAAACTGCTTCCTCGTCATATCCTGTTTTTATCGGACAAGGTATCAGAAAAAGGCTTGCGAAC
TATTAACCTCTTTAAACAGGCCTTTAACAAGGATTATGTTTGTCACGGATGAAGAAGTGGACCGCCTGTATGGTGATGAAAT
GCTCCATTTGCTGCAAGAAAAATGGCCTGTGAAAAAAGTGACGGTACCGAGTGGAGAGCAGGCAAAATCCATGGACATGTAC
ACAAAATTGCAGAGTGAAGCGATTCGTTTTCATATGGATCGCTCCTCATGCATCATCGCATTCGGTGGCGGTGTTGTAGGGG
ATCTTGCCGGTTTTGTGGCTGCCACTTTTATGCGCGGCATTGATTTCATCCAAATGCCTACGACGCTCTTGGCGCATGACAG
CGCAGTCGGAGGAAAAGTAGCCGTGAACCATCCGCTTGGAAAAAATCTAATCGGTGCGTTTTATCAGCCGAAAGCCGTGCTC
TATGATACAGATTTCCTGCGTTCTCTGCCTGAAAAAGAGCTTAGGTCCGGAATGGCAGAAGTGATTAAACACGCTTTTATCT
ATGACAGAGCGTTTCTGGAGGAGCTGCTGAATATCCACTCACTGCGTGATATCACTAATGATCAGCTGAATGACATGATTTT
TAAAGGCATTTCAATTAAAGCGTCTGTCGTTCAGCAGGATGAAAAAGAAGAGGGGATAAGAGCTTACTTAAACTTTGGGCAT
ACGCTCGGCCATGCCGTTGAGGCGGAATATGGATATGGGCAGATCACTCACGGTGATGCCGTAGCCCTCGGAATGCAGTTTG
CTTTATATATAAGCGAAAAGACTGTAGGCTGTGAAATGGACAGAAAACGTTTGGTCAGCTGGCTAAAAAGCCTGGGTTATCC
AAGTCAAATCAGAAAAGAGACGGAAACTTCGGTTCTCTTGAACCGTATGATGAATGATAAGAAAACCCGTGGCGGGAAGATT
CAGTTTATTGTGCTCAACGAATTAGGGAAAGTTGCTGATCATACATTTTCCAGAAATGAACTTGAGAGCTGGCTGAACAAAT
GGCGATTGGAGGAGACATCatgaTCACACTGGCTCACCTTCGGGTGGGCCTTTCTGCGTTTATATACTAGAGAGAGAATATA
AAAAGCCAGATTATTAATCCGGCTTTTTTATTATTTAGGCAACTGAAACGATTCGGATCCTGTATTACTATTCTTAgttaag
atggcaagcttgacaagtatttccgacacatttacaatgaagttGGAGAAAAGATTAACTAATAAGGAGGACAAACATGAGA
GTCCTGATCGTCGATAACTATGACAGCTTTACATTTAACCTGGCGACGTATGTCGAAGAAGTTACAGGCCAAGCACCGACAG
TTGTTAGAAACGATGATATTATCGATGAAACGCTGTTTGATGCGGTTATTCTGTCACCTGGACCGGGACATCCTGGCGTTCT
GGCAGATTTTGGCATTTGCACAGGCATTATTGAAAGAGCACAAGTTCCGATTCTGGGCGTTTGCCTGGGCCATCAAGGCATT
GCACTGGCACATGGCGCAAGAGTTGAACTGGCACCGACACCGGTTCATGGCCAAGTTTCAACAATTTCACATAATGACAGCG
CACTGTTTGACGCAATTCCGAGAGATTTTGATGTCGTCAGATATCATTCAATGATTGCATCAGATCTGCCGGATTCAGTTGA
AGCAACAGCATGGACAGCAGATGGCCTGATTATGGCACTGCAACATAAAACACTGCCGCAATGGGGAGTTCAATTTCATCCG
GAATCAATTGGCGGACAATGGGGACATCAGATTATTCGCAATTTTCTTCATGCAGCACGCAGCTATCATTGGGAAATTCAAG
AAGAAGTTCTGGAAATTTCAGTCGATCCGGCAAGAGTTTTTGCAACACTGTATGGCGCAGCAGAACAAGCATTTTGGCTGGA
TGATGCAGCAGGCACATCATATCTGGGAGATGCATCAGGACCGCTGGCAAGAACAAAAACATTTAGAGTTGGCGAAGGCGAC
TTTTTTGAATGGCTGGCAGCAGATCTGGCAAAAAATACAGTTGCACCTGGCGAAGGATTTAGACTTGGCTGGGTTGGCTATG
TTGGATATGAACTGAAAGCAGAATGCGGAGCACAAGCAGAACATAGAAGCAAACTGCCGGATGCACATCTGATTTTTGCAGA
TAGAGCACTGGCGATTGAAAAAGATAGAGTTAGACTGCTGTCACTGCAAGCAGATGCACAATGGTCAGCACAAGTCGAAGCA
GCACTGAAACAACTGCAACCGGCACCGGCAGCACAAATCAAACCGATTGAACTGCAAGTTAGAGATTCTCGCGAACAGTATC
TGGATAAAATTGCAAAAGCGCAAGATCTGATTAGACGCGGAGAATCATATGAAATTTGCCTGACAACACAACTGTCAGGCGA
ATGCACACAAGATCCGTTTGAACTGTATCTGGCACTGAGAGCAGAAAATCCGACAGCATATGGCTCATTTCTGAAATTTGGC
GAAACAGCAATTCTGTCATCATCACCGGAACGCTTTATTACAATTGATGCAGGCGGAAGAGTCGAATCAAAACCGATCAAAG
GCACAAGAGGCAGAGGCAAAAATGCAGCAGAAGATGCAGAAATCATTAAAGAACTGCAGAGCAATCCGAAAGATCGCGCTGA
AAATCTGATGATTGTTGATCTGGTTAGAAATGATCTGGCGAGAGGCGCACAACCGATTACAGTTAAAACAGAAAAACTGTTC
GACGTGGAAACATTTGCAACAGTTCATCAACTGGTTTCAACAGTTAGCGCACAACTGGGCGAAAAAAATGCAATTGGCTGCA
TTAGAGCAGCATTTCCAGGCGGATCAATGACAGGCGCTCCGAAACTGAGAACAATGGAAATTATTGATGCACTGGAAGCGGC
ACCGAGAGGCATTTATTCAGGCGGACTGGGCTATTTTTCACTGGATGGCTCAGTTGATCTGAGCATGGTTATTAGAACACTG
GTTCTGCATGCTGGCCATCTGGAATATGGCGTTGGCGGAGCAATTCTTGCACTGTCAGATCCGGCTGAAGAATGGGAAGAAA
TTCGCATTAAATCAACACCGCTGCTGAAACTGTTTGGCGTCGAATTTCCGTAATAAACTTTATCTGAGAATAGTCAATCTTC
GGAAATCCCAGGTGGCTCTTAAGGAGGATTTTAGAATGTATTGGATTAATGGCAAACCGTGCAATCAACTGCCGGTTAATGA
TAGAGCAGTTCAATTTGGAGATGGCTGCTTTACAACAATTAGAGTTGAACAAGGCCAAGCAGCACTGCTGCCGCTGCATATT
AAACGCCTGCAAAAAGGCGTTGAAAAACTGTTTATGCCTGCACTGGATTGGCTGCAACTGGAAGATCATATCAAACAAGTTG
CAACAGGCTGCGAATCAGGCGTTCTGAAAGTTATTCTGTCAAGAGGCGTTGGCGGAAGAGGCTATGGCATTTCAGATGCAAT
TGAACCGAATCAAGTTCTGTCATTATCAAGCTATCCGGAACAGTATGTCATTCAACGCAAAAATGGCATTAGCCTGGTTCTG
TCACCGATTGTTATGGGCATTAATCCGCATCTGGCTGGCATTAAACATCTGAATAGACTGGAACAGGTTCTGATCAAACGCT
TTATTGAACAAAGCAAAGCGGATGAAGCACTGGTTCTGGATTCAGATGGCCTGCTGGTTGAATGCTGCACAGCAAACATTTT
TTGGCGCAAAGGCAAAAATGTCTATACACCGGATCTGAATCAATGCGGAGTTGAAGGCGTTATGAGACAGAAAATTATGCAA
CTGCTGGCGGAATCAGATTATAACCTGTCATGCGTCATGAGATATCCGGAAGTTCTGGCACATGCAGATGAAGTCATTATTT
GCAATTCACTGATGCCGGTTATTGCGGTCAATCAAATTCAAGCGCATAAAAATCAACCGGCATGGAAATATCAATCACGCGA
ACTGCATGAATATCTGCTTCCGGCATGCCTGAGACTGTAATAATAAAAGTCTCGTAAAGCGTTCTATCAATAACCCGTTGGT
GCCAGGCATCAAATAAAACGAAAGGCTCAGTCGAAAGACTGGGCCTTTCGTTTTATCTGTTGTTTGTCGGTGAACGCTCTCT
ACTAGAGTCACACTGGCTCACCTTCGGGTGGGCCTTTCTGCGTTTATAgcaggcgagaaaggagagagaacgcaaggagagg
cacgcgagggaggaaaggcaggataccgttcgtatagcatacattatacgaagttatgaattccgaaaaacggttgcattt
aaatcttacatatgtaatactttcaaagactactagtacataaggaggaactact**ATGAACGAGAAAAATATAAAACACAGT**
CAAAACTTTATTACTTCAAAACATAATATAGATAAAATAATGACAAATATAAGATTAAATGAACATGATAATATCTTTGAAA
TCGGCTCAGGAAAAGGCCATTTTACCCTTGAATTAGTAAAGAGGTGTAATTTCGTAACTGCCATTGAAATAGACCATAAATT

ATGCAAAACTACAGAAAATAAACTTGTTGATCACGATAATTTCCAAGTTTTAAACAAGGATATATTGCAGTTTAAATTTCCT
AAAAACCAATCCTATAAAATATATGGTAATATACCTTATAACATAAGTACGGATATAATACGCAAAATTGTTTTTGATAGTA
TAGCTAATGAGATTTATTTAATCGTGGAATACGGGTTTGCTAAAAGATTATTAAATACAAAACGCTCATTGGCATTACTTTT
AATGGCAGAAGTTGATATTTCTATATTAAGTATGGTTCCAAGAGAATATTTTCATCCTAAACCTAAAGTGAATAGCTCACTT
ATCAGATTAAGTAGAAAAAAATCAAGAATATCACACAAAGATAAACAAAAGTATAATTATTTCGTTATGAAATGGGTTAACA
AAGAATACAAGAAAATATTTACAAAAAATCAATTTAACAATTCCTTAAAACATGCAGGAATTGACGATTTAAACAATATTAG
CTTTGAACAATTCTTATCTCTTTTCAATAGCTATAAATTATTTAATAAGTAATAGggggatcttctcgagataacttcgtat
agcatacattatacgaacggtagagagagcacagatacggcgacgacaccgaagcagagcgaagcagtgacaggagcctcgT
ACGATAAGAACAGCTTAGAAATACACAAGAGTGTGTATAAAGCAATTAGAATGAGTTGAGTTAGAGAATAGGGTAGCAGAGA
ATGAGTTTAGTTGAGCTGAGACATTATGTTTATTCTACCCAAAAGAAGTCTTTCTTTTGGGTTTATTTGTTATATAGTATTT
TATCCTCTCATGCCATCTTCTCATTCTCCTTGCCATAAGGAGTGAGAGCAatgAATTTCCAATCAAACATTTCCGCATTTTT
AGAGGACAGCTTGTCCCACCACACGATACCGATTGTGGAGACCTTCACAGTCGATACACTGACACCCATTCAAATGATAGAG
AAGCTTGACAGGGAGATTACGTATCTTCTTGAAAGCAAGGACGATACATCCACTTGGTCCAGATATTCGTTTATCGGCCTGA
ATCCATTTCTCACAATTAAAGAAGAGCAGGGCCGTTTTTCGGCCGCTGATCAGGACAGCAAATCTCTTTACACAGGAAATGA
ACTAAAAGAAGTGCTGAACTGGATGAATACCACATACAAAATCAAAACACCTGAGCTTGGCATTCCTTTTGTCGGCGGAGCT
GTCGGGTACTTAAGCTATGATATGATCCCGCTGATTGAGCCTTCTGTTCCTTCGCATACCAAAGAAACAGACATGGAAAAGT
GTATGCTGTTTGTTTGCCGGACATTAATTGCGTATGATCATGAAACCAAAAACGTCCACTTTATCCAATATGCAAGGCTCAC
TGGAGAGGAAACAAAAAACGAAAAAATGGATGTATTCCATCAAAATCATCTGGAGCTTCAAAATCTCATTGAAAAAATGATG
GACCAAAAAAACATAAAAGAGCTGTTTCTTTCTGCTGATTCATACAAGACACCCAGCTTTGAGACAGTATCTTCTAATTATG
AAAAATCGGCTTTTATGGCTGATGTAGAAAAAATCAAAAGCTATATAAAAGCAGGCGATATCTTCCAGGGTGTTTTATCACA
AAAATTTGAGGTGCCGATAAAAGCAGATGCTTTTGAGTTATACCGAGTGCTTAGGATCGTCAATCCTTCGCCGTATATGTAT
TATATGAAACTGCTAGACAGAGAAATAGTCGGCAGCTCTCCGGAACGGTTAATACACGTTCAAGACGGGCACTTAGAAATCC
ATCCGATTGCCGGTACGAGAAAACGCGGTGCAGACAAAGCTGAAGATGAGAGACTGAAGGTTGAGCTCATGAAGGATGAAAA
AGAAAAAGCGGAGCATTACATGCTCGTTGATCTTGCCCGAAACGATATCGGCAGAGTAGCAGAGTATGGTTCTGTTTCTGTG
CCGGAGTTCACAAAAATTGTTTCCTTTTCACATGTCATGCACATTATCTCGGTGGTTACAGGCCGATTGAAAAAAGGGGTTC
ATCCTGTCGATGCACTGATGTCTGCTTTCCCGGCGGGGACTTTAACAGGCGCACCCAAAATCCGTGCCATGCAGCTTTTGCA
AGAACTCGAGCCAACACCGAGAGAGACATACGGAGGGTGTATTGCCTACATTGGGTTTGACGGGAATATCGACTCTTGTATT
ACGATTCGCACGATGAGTGTAAAGAACGGTGTTGCATCGATACAGGCAGGTGCTGGCATTGTTGCTGATTCTGTTCCGGAAG
CCGAATACGAAGAAAGCTGTAATAAAGCCGGTGCGCTGCTGAAAACGATTCATATTGCAGAAGACATGTTTCATAGCAAGGA
GGATAAAGCTGATGAACAGATTTCTACAATTGTGCGTtga

***aroH* locus** of *B. subtilis* 168 after integration of

Vector B (fusion-protein design): aroB_FR-5'OH-T_{B0014}-spacer$_1$-P_{ymdA}-TSS-RBS$_0$-*pabAB*$_{Cca}$-linker-*pabC*$_{Xbo}$-spacer$_3$-T_{B0015}-lox71-**erm**^{R}-lox66-3'OH-trpE_FR

atgAAGACACTGCATGTTCAAACTGCTTCCTCGTCATATCCTGTTTTTATCGGACAAGGTATCAGAAAAAAGGCTTGCGAAC
TATTAACCTCTTTAAACAGGCCTTTAACAAGGATTATGTTGTCACGGATGAAGAAGTGGACCGCCTGTATGGTGATGAAAT
GCTCCATTTGCTGCAAGAAAAATGGCCTGTGAAAAAAGTGACGGTACCGAGTGGAGAGCAGGCAAAATCCATGGACATGTAC
ACAAAATTGCAGAGTGAAGCGATTCGTTTTCATATGGATCGCTCCTCATGCATCATCGCATTCGGTGGCGGTGTTGTAGGGG
ATCTTGCCGGTTTTGTGGCTGCCACTTTTATGCGCGGCATTGATTTCATCCAAATGCCTACGACGCTCTTGGCGCATGACAG
CGCAGTCGGAGGAAAAGTAGCCGTGAACCATCCGCTTGGAAAAAATCTAATCGGTGCGTTTTATCAGCCGAAAGCCGTGCTC
TATGATACAGATTTCCTGCGTTCTCTGCCTGAAAAAGAGCTTAGGTCCGGAATGGCAGAAGTGATTAAACACGCTTTTATCT
ATGACAGAGCGTTTCTGGAGGAGCTGCTGAATATCCACTCACTGCGTGATATCACTAATGATCAGCTGAATGACATGATTTT
TAAAGGCATTTCAATTAAAGCGTCTGTCGTTCAGCAGGATGAAAAAGAAGAGGGGATAAGAGCTTACTTAAACTTTGGGCAT
ACGCTCGGCCATGCCGTTGAGGCGGAATATGGATATGGGCAGATCACTCACGGTGATGCCGTAGCCCTCGGAATGCAGTTTG
CTTTATATATAAGCGAAAAGACTGTAGGCTGTGAAATGGACAGAAAACGTTTGGTCAGCTGGCTAAAAAGCCTGGGTTATCC
AAGTCAAATCAGAAAAGAGACGGAAACTTCGGTTCTCTTGAACCGTATGATGAATGATAAGAAAACCCGTGGCGGGAAGATT
CAGTTTATTGTGCTCAACGAATTAGGGAAAGTTGCTGATCATACATTTTCCAGAAATGAACTTGAGAGCTGGCTGAACAAAT
GGCGATTGGAGGAGACATCatgaTCACACTGGCTCACCTTCGGGTGGGCCTTTCTGCGTTTATATACTAGAGAGAGAATATA
AAAAGCCAGATTATTAATCCGGCTTTTTTATTATTTAGGCAACTGAAACGATTCGGATCCTGTATTACTATTCTTAgttaag
atggcaagcttgacaagtatttccgacacatttacaatgaagttGGAGAAAAGATTAACTAATAAGGAGGACAAACATGAGA
GTCCTGATCGTCGATAACTATGACAGCTTTACATTTAACCTGGCGACGTATGTCGAAGAAGTTACAGGCCAAGCACCGACAG
TTGTTAGAAACGATGATATTATCGATGAAACGCTGTTTGATGCGGTTATTCTGTCACCTGGACCGGGACATCCTGGCGTTCT
GGCAGATTTTGGCATTTGCACAGGCATTATTGAAAGAGCACAAGTTCCGATTCTGGGCGTTTGCCTGGGCCATCAAGGCATT
GCACTGGCACATGGCGCAAGAGTTGAACTGGCACCGACACCGGTTCATGGCCAAGTTTCAACAATTTCACATAATGACAGCG
CACTGTTTGACGCAATTCCGAGAGATTTTGATGTCGTCAGATATCATTCAATGATTGCATCAGATCTGCCGGATTCAGTTGA
AGCAACAGCATGGACAGCAGATGGCCTGATTATGGCACTGCAACATAAAACACTGCCGCAATGGGGAGTTCAATTTCATCCG
GAATCAATTGGCGGACAATGGGGACATCAGATTATTCGCAATTTTCTTCATGCAGCACGCAGCTATCATTGGGAAATTCAAG
AAGAAGTTCTGGAAATTTCAGTCGATCCGGCAAGAGTTTTTGCAACACTGTATGGCGCAGCAGAACAAGCATTTTGGCTGGA
TGATGCAGCAGGCACATCATATCTGGGAGATGCATCAGGACCGCTGGCAAGAACAAAAACATTTAGAGTTGGCGAAGGCGAC
TTTTTTGAATGGCTGGCAGCAGATCTGGCAAAAAATACAGTTGCACCTGGCGAAGGATTTAGACTTGGCTGGGTTGGCTATG
TTGGATATGAACTGAAAGCAGAATGCGGAGCACAAGCAGAACATAGAAGCAAACTGCCGGATGCACATCTGATTTTTGCAGA
TAGAGCACTGGCGATTGAAAAAGATAGAGTTAGACTGCTGTCACTGCAAGCAGATGCACAATGGTCAGCACAAGTCGAAGCA
GCACTGAAACAACTGCAACCGGCACCGGCAGCACAAATCAAACCGATTGAACTGCAAGTTAGAGATTCTCGCGAACAGTATC
TGGATAAAATTGCAAAAGCGCAAGATCTGATTAGACGCGGAGAATCATATGAAATTTGCCTGACAACACAACTGTCAGGCGA
ATGCACACAAGATCCGTTTGAACTGTATCTGGCACTGAGAGCAGAAAATCCGACAGCATATGGCTCATTTCTGAAATTTGGC
GAAACAGCAATTCTGTCATCATCACCGGAACGCTTTATTACAATTGATGCAGGCGGAAGAGTCGAATCAAAACCGATCAAAG
GCACAAGAGGCAGAGGCAAAAATGCAGCAGAAGATGCAGAAATCATTAAAGAACTGCAGAGCAATCCGAAAGATCGCGCTGA
AAATCTGATGATTGTTGATCTGGTTAGAAATGATCTGGCGAGAGGCGCACAACCGATTACAGTTAAAACAGAAAAACTGTTC
GACGTGGAAACATTTGCAACAGTTCATCAACTGGTTTCAACAGTTAGCGCACAACTGGGCGAAAAAAATGCAATTGGCTGCA
TTAGAGCAGCATTTCCAGGCGGATCAATGACAGGCGCTCCGAAACTGAGAACAATGGAAATTATTGATGCACTGGAAGCGGC
ACCGAGAGGCATTTATTCAGGCGGACTGGGCTATTTTTCACTGGATGGCTCAGTTGATCTGAGCATGGTTATTAGAACACTG
GTTCTGCATGCTGGCCATCTGGAATATGGCGTTGGCGGAGCAATTCTTGCACTGTCAGATCCGGCTGAAGAATGGGAAGAAA
TTCGCATTAAATCAACACCGCTGCTGAAACTGTTTGGCGTCGAATTTCCTGGTGGATCAGGTGGAGGTGGATCAGGTGGAAT
GTATTGGATTAATGGCAAACCGTGCAATCAACTGCCGGTTAATGATAGAGCAGTCCAATTTGGAGATGGCTGCTTTACAACA
ATTAGAGTCGAACAAGGCCAAGCGGCACTGCTGCCGCTGCATATTAAACGCCTGCAAAAAGGCGTTGAAAAGTTATTTATGC
CTGCACTGGATTGGCTTCAGCTGGAAGATCATATCAAACAAGTTGCAACAGGCTGCGAATCAGGCGTTCTGAAAGTTATTCT
TTCAAGAGGCGTTGGAGGCCGTGGCTATGGCATTTCAGATGCAATTGAACCGAATCAAGTTCTGTCATTAAGCAGCTATCCG
GAACAATATGTCATTCAACGCAAAAATGGCATTAGCCTGGTTCTTTCACCGATTGTTATGGGCATTAATCCGCATCTGGCAG
GCATTAAACATCTGAATAGACTGGAACAGGTTCTGATCAAACGGTTTATCGAACAAAGCAAAGCAGATGAAGCACTGGTCCT
GGATTCAGATGGACTGCTGGTTGAATGCTGCACAGCAAACATTTTTTGGCGCAAAGGCAAAAACGTTTATACACCGGATCTT
AATCAATGCGGAGTTGAAGGCGTTATGCGCCAGAAAATTATGCAACTGCTGGCAGAAAGCGATTATAACCTGTCATGCGTTA
TGAGATATCCGGAAGTTCTTGCACATGCGGATGAAGTCATTATTTGCAATTCACTGATGCCGGTCATTGCGGTCAATCAAAT
TCAAGCGCATAAAAATCAACCGGCATGGAAATATCAATCACGCGAACTGCATGAATATCTGCTTCCGGCATGCCTGAGACTG
TAATAATAAAAGTCTCGTAAAGCGTTCTATCAATAACCCGTTGGTGCCAGGCATCAAATAAAACGAAAGGCTCAGTCGAAAG
ACTGGGCCTTTCGTTTTATCTGTTGTTTGTCGGTGAACGCTCTCTACTAGAGTCACACTGGCTCACCTTCGGGTGGGCCTTT
CTGCGTTTATAgcaggcgagaaaggagagagaacgcaaggagaggcacgcgagggaggaaaggcaggataccgttcgtatag
catacattatacgaagttatgaattccgaaaaaacggttgcatttaaatcttacatatgtaatactttcaaagactactagt
acataaggaggaactact**ATGAACGAGAAAAATATAAAACACAGTCAAAACTTTATTACTTCAAAACATAATATAGATAAAA**
TAATGACAAATATAAGATTAAATGAACATGATAATATCTTTGAAATCGGCTCAGGAAAAGGCCATTTTACCCTTGAATTAGT
AAAGAGGTGTAATTTCGTAACTGCCATTGAAATAGACCATAAATTATGCAAAACTACAGAAAATAAACTTGTTGATCACGAT

AATTTCCAAGTTTTAAACAAGGATATATTGCAGTTTAAATTTCCTAAAAACCAATCCTATAAAATATATGGTAATATACCTT
ATAACATAAGTACGGATATAATACGCAAAATTGTTTTTGATAGTATAGCTAATGAGATTTATTTAATCGTGGAATACGGGTT
TGCTAAAAGATTATTAAATACAAAACGCTCATTGGCATTACTTTTAATGGCAGAAGTTGATATTTCTATATTAAGTATGGTT
CCAAGAGAATATTTTCATCCTAAACCTAAAGTGAATAGCTCACTTATCAGATTAAGTAGAAAAAAATCAAGAATATCACACA
AAGATAAACAAAAGTATAATTATTTCGTTATGAAATGGGTTAACAAAGAATACAAGAAAATATTTACAAAAAATCAATTTAA
CAATTCCTTAAAACATGCAGGAATTGACGATTTAAACAATATTAGCTTTGAACAATTCTTATCTCTTTTCAATAGCTATAAA
TTATTTAATAAGTAATAGggggatcttctcgagataacttcgtatagcatacattatacgaacggtagagagagcacagata
cggcgacgacaccgaagcagagcgaagcagtgacaggagcctcgTACGATAAGAACAGCTTAGAAATACACAAGAGTGTGTA
TAAAGCAATTAGAATGAGTTGAGTTAGAGAATAGGGTAGCAGAGAATGAGTTTAGTTGAGCTGAGACATTATGTTTATTCTA
CCCAAAAGAAGTCTTTCTTTTGGGTTTATTTGTTATATAGTATTTTATCCTCTCATGCCATCTTCTCATTCTCCTTGCCATA
AGGAGTGAGAGCAatgAATTTCCAATCAAACATTTCCGCATTTTTAGAGGACAGCTTGTCCCACCACACGATACCGATTGTG
GAGACCTTCACAGTCGATACACTGACACCCATTCAAATGATAGAGAAGCTTGACAGGGAGATTACGTATCTTCTTGAAAGCA
AGGACGATACATCCACTTGGTCCAGATATTCGTTTATCGGCCTGAATCCATTTCTCACAATTAAAGAAGAGCAGGGCCGTTT
TTCGGCCGCTGATCAGGACAGCAAATCTCTTTACACAGGAAATGAACTAAAAGAAGTGCTGAACTGGATGAATACCACATAC
AAAATCAAAACACCTGAGCTTGGCATTCCTTTTGTCGGCGGAGCTGTCGGGTACTTAAGCTATGATATGATCCCGCTGATTG
AGCCTTCTGTTCCTTCGCATACCAAAGAAACAGACATGGAAAAGTGTATGCTGTTTGTTTGCCGGACATTAATTGCGTATGA
TCATGAAACCAAAAACGTCCACTTTATCCAATATGCAAGGCTCACTGGAGAGGAAACAAAAAACGAAAAAATGGATGTATTC
CATCAAAATCATCTGGAGCTTCAAAATCTCATTGAAAAAATGATGGACCAAAAAAACATAAAAGAGCTGTTTCTTTCTGCTG
ATTCATACAAGACACCCAGCTTTGAGACAGTATCTTCTAATTATGAAAAATCGGCTTTTATGGCTGATGTAGAAAAAATCAA
AAGCTATATAAAAGCAGGCGATATCTTCCAGGGTGTTTTATCACAAAAATTTGAGGTGCCGATAAAAGCAGATGCTTTTGAG
TTATACCGAGTGCTTAGGATCGTCAATCCTTCGCCGTATATGTATTATATGAAACTGCTAGACAGAGAAATAGTCGGCAGCT
CTCCGGAACGGTTAATACACGTTCAAGACGGGCACTTAGAAATCCATCCGATTGCCGGTACGAGAAAACGCGGTGCAGACAA
AGCTGAAGATGAGAGACTGAAGGTTGAGCTCATGAAGGATGAAAAAGAAAAAGCGGAGCATTACATGCTCGTTGATCTTGCC
CGAAACGATATCGGCAGAGTAGCAGAGTATGGTTCTGTTTCTGTGCCGGAGTTCACAAAAATTGTTTCCTTTTCACATGTCA
TGCACATTATCTCGGTGGTTACAGGCCGATTGAAAAAAGGGGTTCATCCTGTCGATGCACTGATGTCTGCTTTCCCGGCGGG
GACTTTAACAGGCGCACCCAAAATCCGTGCCATGCAGCTTTTGCAAGAACTCGAGCCAACACCGAGAGAGACATACGGAGGG
TGTATTGCCTACATTGGGTTTGACGGAATATCGACTCTTGTATTACGATTCGCACGATGAGTGTAAAGAACGGTGTTGCAT
CGATACAGGCAGGTGCTGGCATTGTTGCTGATTCTGTTCCGGAAGCCGAATACGAAGAAAGCTGTAATAAAGCCGGTGCGCT
GCTGAAAACGATTCATATTGCAGAAGACATGTTTCATAGCAAGGAGGATAAAGCTGATGAACAGATTTCTACAATTGTGCGT
tga

Construction of vector A

Primers:

frag 2 for (pabAB): TCACACTGGCTCACCTTCGGGTG (T_m = 60°C)
frag 2 rev (pabAB): AGGAAATTCGACGCCAAACAGTTTCAG (T_m = 60°C)
pabC for: GAATGTATTGGATTAATGGCAAACCGTGC (T_m = 59°C)
frag 4 rev (erm-HI): cgaggctcctgtcactgcttc (T_m = 58.5°C)

frag 1 for (aroB): GACGCTCTTGGCGCATGACAGC (T_m = 62°C)
frag 1 for (pUC19-aroB): aattcgagctcggtacccggggatcctctagaGACGCTCTTGGCGCAT (T_m = 52°C / 76.2°C)
frag 1 rev (pabAB - aroB): ATAAACGCAGAAAGGCCCACCCGAAGGTGAGCCAGTGTGAtcatGATGTCTCCTCCAATC (T_m = 49°C / 75.5°C)

frag 5 for (trpE - ermHI): acaccgaagcagagcgaagcagtgacaggagcctcgTACGATAAGAACAGCTTA (T_m = 43°C / 76.5°C)
frag 5 rev (aroH): TCATGATCATACGCAATTAATGTCCGGC (T_m = 60°C)
frag 5 rev (pUC19-trpE): accatgattacgccaagcttgcatgcctgcaggtcgacTCATGATCATACGCAATTAA (T_m = 46°C / 75.7°C)

pUC19 for (trpE-MCS): ttgtttgccggacattaattgcgtatgatcatgaGTCGACCTGCAGGCATGC (T_m = 55°C / 74°C)
pUC19 rev (aroB-MCS): gactgcgctgtcatgcgccaagagcgtcTCTAGAGGATCCCCGGGT (T_m = 51°C / 77°C)

PCR mix:

Fragment 1 frag 1 for & frag 1 rev / Fragment 5 frag 5 for & frag 5 rev	2 × 5 µL primer (10 µM, ≙ 0.5 µM final), 1 µL template (≈ 35 ng gDNA), 4 µL DMSO, 50 µL Q5 master-mix, 35 µL H_2O, (100 µL final)
$pUC19_{aroH\_FR}$ frag 1 for (pUC19-aroB) & frag 5 rev (pUC19-trpE)	2 × 2.5 µL primer (10 µM, ≙ 0.5 µM final), 1 µL template (≈ 10 pg pUC19), 1 µL DMSO, 25 µL Q5 master-mix, 17 µL H_2O, (50 µL final)

PCR conditions:

Fragment 1	30 sec @ 98°C – 34× (10 sec @ 98°C, 20 sec @ **49**°C**+0.7**°C /cycle, 20 sec @ 72°C) – 2 min @ 72°C – hold @ 4°C
Fragment 5	30 sec @ 98°C – 34× (10 sec @ 98°C, 20 sec @ **43**°C**+0.9**°C /cycle, 20 sec @ 72°C) – 2 min @ 72°C – hold @ 4°C
$pUC19_{aroH\_FR}$	20 sec @ 98°C – 32× (10 sec @ 98°C, 20 sec @ **72**°C**-0.4**°C /cycle, 2 min @ 72°C) – 4 min @ 72°C – hold @ 4°C

Assembly mix:

Fragment	amount each
Fragment 1	91 ng
Fragment 2	276.1 ng
Fragment 3+4	269.6 ng
Fragment 5	91 ng
pUC19 backbone	357.2 ng

0.2 pmol each fragment + 10 µL NEBuilder ➔ 20 µL total volume

- ➔ primers for sequencing of vector A: frag 2 for, frag 2 rev, frag 3 for

Construction of vector B

PCR mix:

F2-F1-pUC19-F5-F3+4 frag 2 rev (pabAB) & pabC for	2 × 10 µL primer (10 µM, ≙ 1 µM final), 1 µL template (≈ 100 ng pUC19-A), 4 µL DMSO, 50 µL Q5 master-mix, 25 µL H_2O, (100 µL final)

PCR conditions:

F2-F1-pUC19-F5-F3+4	30 sec @ 98°C – 32× (10 sec @ 98°C, 20 sec @ **72**°C-**0.4**°C /cycle, 6 min @ 72°C) – 12 min @ 72°C – hold @ 4°C

Assembly mix:

Fragment	amount each
F3+4-F5-pUC19-F1-F2	106.78 ng
linker	3.38 ng

0.04 pmol backbone, 0.08 pmol linker + 10 µL NEBuilder ➔ 20 µL total volume

➔ primers for sequencing of vector B: verB2, verC2 (frag2 rev, frag3 for)

verC2: CACTGGATGGCTCAGTTGAT (52°C)
verB2: TGCAGGCGTTTAATATGCAG (53°C)

Integration and verification of synthetic operons

Primers:

frag 1 for (aroB): GACGCTCTTGGCGCATGACAGC (T_m = 62°C)
frag 5 rev (aroH): TCATGATCATACGCAATTAATGTCCGGC (T_m = 60°C)

verB (rev, pabAB): CCAGATATGATGTGCCTGCTGC (T_m = 57.4°C)
verC (for, pabC): GGTCAATCAAATTCAAGCGC (T_m = 51°C)

verA (for, aroB): TGCAGAGTGAAGCGATTCGT (T_m = 55.6°C)
verD (rev, trpE): CTCTCCAGTGAGCCTTGC (T_m = 52.4°C)

PCR mix:

Vector A/B	2 × 5 µL primer (10 µM, ≙ 0.5 µM final), 1 µL template (≈ 100 ng pUC19-A/B), 4 µL DMSO, 50 µL Q5 master-mix, 36 µL H_2O, (100 µL final)
"out-in" / "in-out"	2 × 5 µL primer (10 µM, ≙ 0.5 µM final), 1 µL template (≈ 35 ng gDNA), 4 µL DMSO, 50 µL Q5 master-mix, 36 µL H_2O, (100 µL final)
"locus spanning"	2 × 10 µL primer (10 µM, ≙ 1 µM final), 1 µL template (≈ 35 ng gDNA), 4 µL DMSO, 50 µL Q5 master-mix, 26 µL H_2O, (100 µL final)

PCR conditions:

Vector A/B frag 1 for & frag 5 rev	20 sec @ 98°C – 32× (10 sec @ 98°C, 20 sec @ **70**°C-**0.3**°C /cycle, 4 min @ 72°C) – 8 min @ 72°C – hold @ 4°C
"out-in" verA-verB	30 sec @ 98°C – 48× (10 sec @ 98°C, 20 sec @ **58**°C-**0.2**°C /cycle, 2 min @ 72°C) – 4 min @ 72°C – hold @ 4°C
"in-out" verC-verD	30 sec @ 98°C – 48× (10 sec @ 98°C, 20 sec @ **53**°C-**0.2**°C /cycle, 2 min @ 72°C) – 4 min @ 72°C – hold @ 4°C
"locus spanning" verA-verD	30 sec @ 98°C – 48× (10 sec @ 98°C, 20 sec @ **56**°C-**0.2**°C /cycle, 4 min @ 72°C) – 8 min @ 72°C – hold @ 4°C

<u>Construction of vector A^{tagged}</u>

- add Lumio™-tag to C-terminal ends of *pabAB* and *pabC* (flanked by spacer sequences of 2×Gly-Ser / 2×Gly)

Spacer	Gly Ser Gly Ser Gly
	GGA TCA GGA TCA
Lumio™	Cys Cys Pro Gly Cys Cys
	TGC TGC CCG GGA TGC TGC
Spacer	Gly Gly
	GGA GGC

insertion sequence: GGATCAGGATCA**TGCTGCCCGGGATGCTGC**GGAGGC

linker fragment pabAB (gBlock5):
ATTAAATCAACACcgctgctgaaactgtttggcgtcgaatttccgGGATCAGGATCA**TGCTGCCCGGGATGCTGC**GGAGGCtaat
aaactttatctgagaatagtcaatcttcGGAAATCCCAGGT

linker fragment pabC (gBlock6):
TCACGCGAACTGCatgaatatctgcttccggcatgcctgagactgGGATCAGGATCA**TGCTGCCCGGGATGCTGC**GGAGGCtaa
taataaaagtctcgtaaagcgttctatcaATAACCCGTTGGT

pabAB linker-tag for: Ctaataaactttatctgagaatagtc (T_m = 47.6 / 49.5°C)
pabAB linker-tag rev: cggaaattcgacgccaaac (T_m = 52.35°C)
pabC linker-tag for: Ctaataataaaagtctcgtaaagc (T_m = 47.5 / 49.4°C)
pabC linker-tag rev: cagtctcaggcatgccg (T_m = 52.66°C)

amplicon A1 “pabAB linker-tag rev – pabC linker-tag for”: 7200 bp
amplicon A2 “pabC linker-tag rev – pabAB linker-tag for”: 900 bp

<u>Vector A^{tagged}:</u> 5‘OH-T_{B0014}-spacer$_1$-P_{ymdA}-TSS-RBS_0-*$pabAB_{Cca}$*-tag-spacer$_2$-RBS_1-*$pabC_{Xbo}$*-tag-spacer$_3$-T_{B0015}-lox71-**erm^R**-lox66-3’OH
GACGCTCTTGGCGCATGACAGCGCAGTCGGAGGAAAAGTAGCCGTGAACCATCCGCTTGGAAAAAATCTAATCGGTGCGTTT
TATCAGCCGAAAGCCGTGCTCTATGATACAGATTTCCTGCGTTCTCTGCCTGAAAAAGAGCTTAGGTCCGGAATGGCAGAAG
TGATTAAACACGCTTTTATCTATGACAGAGCGTTTCTGGAGGAGCTGCTGAATATCCACTCACTGCGTGATATCACTAATGA
TCAGCTGAATGACATGATTTTTAAAGGCATTTCAATTAAAGCGTCTGTCGTTCAGCAGGATGAAAAAGAAGAGGGGATAAGA
GCTTACTTAAACTTTGGGCATACGCTCGGCCATGCCGTTGAGGCGGAATATGGATATGGGCAGATCACTCACGGTGATGCCG
TAGCCCTCGGAATGCAGTTTGCTTTATATATAAGCGAAAAGACTGTAGGCTGTGAAATGGACAGAAAACGTTTGGTCAGCTG
GCTAAAAAGCCTGGGTTATCCAAGTCAAATCAGAAAAGAGACGGAAACTTCGGTTCTCTTGAACCGTATGATGAATGATAAG
AAAACCCGTGGCGGGAAGATTCAGTTTATTGTGCTCAACGAATTAGGGAAAGTTGCTGATCATACATTTTCCAGAAATGAAC
TTGAGAGCTGGCTGAACAAATGGCGATTGGAGGAGACATCatgaTCACACTGGCTCACCTTCGGGTGGGCCTTTCTGCGTTT
ATATACTAGAGAGAGAATATAAAAAGCCAGATTATTAATCCGGCTTTTTTATTATTTAGGCAACTGAAACGATTCGGATCCT
GTATTACTATTCTTAgttaagatggcaagcttgacaagtatttccgacacatttacaatgaagttGGAGAAAAGATTAACTA
ATAAGGAGGACAAACATGAGAGTCCTGATCGTCGATAACTATGACAGCTTTACATTTAACCTGGCGACGTATGTCGAAGAAG
TTACAGGCCAAGCACCGACAGTTGTTAGAAACGATGATATTATCGATGAAACGCTGTTTGATGCGGTTATTCTGTCACCTGG
ACCGGGACATCCTGGCGTTCTGGCAGATTTTGGCATTTGCACAGGCATTATTGAAAGAGCACAAGTTCCGATTCTGGGCGTT
TGCCTGGGCCATCAAGGCATTGCACTGGCACATGGCGCAAGAGTTGAACTGGCACCGACACCGGTTCATGGCCAAGTTTCAA
CAATTTCACATAATGACAGCGCACTGTTTGACGCAATTCCGAGAGATTTTGATGTCGTCAGATATCATTCAATGATTGCATC
AGATCTGCCGGATTCAGTTGAAGCAACAGCATGGACAGCAGATGGCCTGATTATGGCACTGCAACATAAAACACTGCCGCAA
TGGGGAGTTCAATTTCATCCGGAATCAATTGGCGGACAATGGGGACATCAGATTATTCGCAATTTTCTTCATGCAGCACGCA
GCTATCATTGGGAAATTCAAGAAGAAGTTCTGGAAATTTCAGTCGATCCGGCAAGAGTTTTTGCAACACTGTATGGCGCAGC
AGAACAAGCATTTTGGCTGGATGATGCAGCAGGCACATCATATCTGGGAGATGCATCAGGACCGCTGGCAAGAACAAAAACA
TTTAGAGTTGGCGAAGGCGACTTTTTTGAATGGCTGGCAGCAGATCTGGCAAAAAATACAGTTGCACCTGGCGAAGGATTTA
GACTTGGCTGGGTTGGCTATGTTGGATATGAACTGAAAGCAGAATGCGGAGCACAAGCAGAACATAGAAGCAAACTGCCGGA

TGCACATCTGATTTTTGCAGATAGAGCACTGGCGATTGAAAAAGATAGAGTTAGACTGCTGTCACTGCAAGCAGATGCACAA
TGGTCAGCACAAGTCGAAGCAGCACTGAAACAACTGCAACCGGCACCGGCAGCACAAATCAAACCGATTGAACTGCAAGTTA
GAGATTCTCGCGAACAGTATCTGGATAAAATTGCAAAAGCGCAAGATCTGATTAGACGCGGAGAATCATATGAAATTTGCCT
GACAACACAACTGTCAGGCGAATGCACACAAGATCCGTTTGAACTGTATCTGGCACTGAGAGCAGAAAATCCGACAGCATAT
GGCTCATTTCTGAAATTTGGCGAAACAGCAATTCTGTCATCATCACCGGAACGCTTTATTACAATTGATGCAGGCGGAAGAG
TCGAATCAAAACCGATCAAAGGCACAAGAGGCAGAGGCAAAAATGCAGCAGAAGATGCAGAAATCATTAAAGAACTGCAGAG
CAATCCGAAAGATCGCGCTGAAAATCTGATGATTGTTGATCTGGTTAGAAATGATCTGGCGAGAGGCGCACAACCGATTACA
GTTAAAACAGAAAAACTGTTCGACGTGGAAACATTTGCAACAGTTCATCAACTGGTTTCAACAGTTAGCGCACAACTGGGCG
AAAAAAATGCAATTGGCTGCATTAGAGCAGCATTTCCAGGCGGATCAATGACAGGCGCTCCGAAACTGAGAACAATGGAAAT
TATTGATGCACTGGAAGCGGCACCGAGAGGCATTTATTCAGGCGGACTGGGCTATTTTTCACTGGATGGCTCAGTTGATCTG
AGCATGGTTATTAGAACACTGGTTCTGCATGCTGGCCATCTGGAATATGGCGTTGGCGGAGCAATTCTTGCACTGTCAGATC
CGGCTGAAGAATGGGAAGAAATTCGCATTAAATCAACACCGCTGCTGAAACTGTTTGGCGTCGAATTTCCGGGATCAGGATC
A**TGCTGCCCGGGATGCTGC**GGAGGCTAATAAACTTTATCTGAGAATAGTCAATCTTCGGAAATCCCAGGTGGCTCTTAAGGA
GGATTTTAGAATGTATTGGATTAATGGCAAACCGTGCAATCAACTGCCGGTTAATGATAGAGCAGTTCAATTTGGAGATGGC
TGCTTTACAACAATTAGAGTTGAACAAGGCCAAGCAGCACTGCTGCCGCTGCATATTAAACGCCTGCAAAAGGCGTTGAAA
AACTGTTTATGCCTGCACTGGATTGGCTGCAACTGGAAGATCATATCAAACAAGTTGCAACAGGCTGCGAATCAGGCGTTCT
GAAAGTTATTCTGTCAAGAGGCGTTGGCGGAAGAGGCTATGGCATTTCAGATGCAATTGAACCGAATCAAGTTCTGTCATTA
TCAAGCTATCCGGAACAGTATGTCATTCAACGCAAAAATGGCATTAGCCTGGTTCTGTCACCGATTGTTATGGGCATTAATC
CGCATCTGGCTGGCATTAAACATCTGAATAGACTGGAACAGGTTCTGATCAAACGCTTTATTGAACAAAGCAAAGCGGATGA
AGCACTGGTTCTGGATTCAGATGGCCTGCTGGTTGAATGCTGCACAGCAAACATTTTTTGGCGCAAAGGCAAAAATGTCTAT
ACACCGGATCTGAATCAATGCGGAGTTGAAGGCGTTATGAGACAGAAAATTATGCAACTGCTGGCGGAATCAGATTATAACC
TGTCATGCGTCATGAGATATCCGGAAGTTCTGGCACATGCAGATGAAGTCATTATTTGCAATTCACTGATGCCGGTTATTGC
GGTCAATCAAATTCAAGCGCATAAAAATCAACCGGCATGGAAATATCAATCACGCGAACTGCATGAATATCTGCTTCCGGCA
TGCCTGAGACTGGGATCAGGATCA**TGCTGCCCGGGATGCTGC**GGAGGCTAATAATAAAAGTCTCGTAAAGCGTTCTATCAAT
AACCCGTTGGTGCCAGGCATCAAATAAAACGAAAGGCTCAGTCGAAAGACTGGGCCTTTCGTTTTATCTGTTGTTTGTCGGT
GAACGCTCTCTACTAGAGTCACACTGGCTCACCTTCGGGTGGGCCTTTCTGCGTTTATAgcaggcgagaaaggagagagaac
gcaaggagaggcacgcgagggaggaaaggcaggataccgttcgtatagcatacattatacgaagttatgaattccgaaaaaa
cggttgcatttaaatcttacatatgtaatactttcaaagactactagtacataaggaggaactact**ATGAACGAGAAAAATA**
TAAAACACAGTCAAAACTTTATTACTTCAAAACATAATATAGATAAAATAATGACAAATATAAGATTAAATGAACATGATAA
TATCTTTGAAATCGGCTCAGGAAAAGGCCATTTTACCCTTGAATTAGTAAAGAGGTGTAATTTCGTAACTGCCATTGAAATA
GACCATAAATTATGCAAAACTACAGAAAATAAACTTGTTGATCACGATAATTTCCAAGTTTTAAACAAGGATATATTGCAGT
TTAAATTTCCTAAAAACCAATCCTATAAAATATATGGTAATATACCTTATAACATAAGTACGGATATAATACGCAAAATTGT
TTTTGATAGTATAGCTAATGAGATTTATTTAATCGTGGAATACGGGTTTGCTAAAAGATTATTAAATACAAAACGCTCATTG
GCATTACTTTTAATGGCAGAAGTTGATATTTCTATATTAAGTATGGTTCCAAGAGAATATTTTCATCCTAAACCTAAAGTGA
ATAGCTCACTTATCAGATTAAGTAGAAAAAAATCAAGAATATCACACAAAGATAAACAAAAGTATAATTATTTCGTTATGAA
ATGGGTTAACAAAGAATACAAGAAAATATTTACAAAAAATCAATTTAACAATTCCTTAAAACATGCAGGAATTGACGATTTA
AACAATATTAGCTTTGAACAATTCTTATCTCTTTTCAATAGCTATAAATTATTTAATAAGTAATAGggggatcttctcgaga
taacttcgtatagcatacattatacgaacggtagagagagcacagatacggcgacgacaccgaagcagagcgaagcagtgac
aggagcctcgTACGATAAGAACAGCTTAGAAATACACAAGAGTGTGTATAAAGCAATTAGAATGAGTTGAGTTAGAGAATAG
GGTAGCAGAGAATGAGTTTAGTTGAGCTGAGACATTATGTTTATTCTACCCAAAAGAAGTCTTTCTTTTGGGTTTATTTGTT
ATATAGTATTTTATCCTCTCATGCCATCTTCTCATTCTCCTTGCCATAAGGAGTGAGAGCAatgAATTTCCAATCAAACATT
TCCGCATTTTTAGAGGACAGCTTGTCCCACCACACGATACCGATTGTGGAGACCTTCACAGTCGATACACTGACACCCATTC
AAATGATAGAGAAGCTTGACAGGGAGATTACGTATCTTCTTGAAAGCAAGGACGATACATCCACTTGGTCCAGATATTCGTT
TATCGGCCTGAATCCATTTCTCACAATTAAAGAAGAGCAGGGCCGTTTTTCGGCCGCTGATCAGGACAGCAAATCTCTTTAC
ACAGGAAATGAACTAAAAGAAGTGCTGAACTGGATGAATACCACATACAAAATCAAAACACCTGAGCTTGGCATTCCTTTTG
TCGGCGGAGCTGTCGGGTACTTAAGCTATGATATGATCCCGCTGATTGAGCCTTCTGTTCCTTCGCATACCAAAGAAACAGA
CATGGAAAAGTGTATGCTGTTTGTTTGCCGGACATTAATTGCGTATGATCATGA

Construction of vector B^{tagged}

- introduce Lumio™-tag in-between *pabAB* – *pabC* (flanked by spacer sequences of 2×Gly-Gly-Ser-Gly-Gly)

Spacer	Gly Gly Ser Gly Gly Gly Gly Ser Gly Gly
	GGT GGA TCA GGT GGA GGT GGA TCA GGT GGA
Lumio™	Cys Cys Pro Gly Cys Cys
	TGC TGC CCG GGA TGC TGC
Spacer	Gly Gly Ser Gly Gly Gly Gly Ser Gly Gly
	GGT GGA TCA GGT GGA GGT GGA TCA GGT GGA

insertion sequence:
GGTGGATCAGGTGGAGGTGGATCAGGTGGA**TGCTGCCCGGGATGCTGC**GGTGGATCAGGTGGAGGTGGAT
CAGGTGGA

linker fragment pabABC (gBlock4):
TTCGCATTAAATCAACACCGCTGCTGAAACTGTTTGGCGTCGAATTTCCTGGAGGATCAGGCGGAGGCGGAAGCGGAGGC**TG
CTGTCCGGGATGCTGC**GGAGGATCTGGTGGCGGAGGCTCTGGCGGAATGTATTGGATTAATGGCAAACCGTGCAATCAACTG
CCGGTTAATGATAG

amplicon B1 "frag 2 rev (pabAB) – pabC for": 8200 bp

Vector B^{tagged}: 5'OH-T$_{B0014}$-spacer$_1$-P$_{ymdA}$-TSS-RBS$_0$-*pabAB*$_{Cca}$-linker-tag-linker-*pabC*$_{Xbo}$-spacer$_3$-T$_{B0015}$-lox71-**erm^R**-lox66-3'OH
GACGCTCTTGGCGCATGACAGCGCAGTCGGAGGAAAAGTAGCCGTGAACCATCCGCTTGGAAAAAATCTAATCGGTGCGTTT
TATCAGCCGAAAGCCGTGCTCTATGATACAGATTTCCTGCGTTCTCTGCCTGAAAAAGAGCTTAGGTCCGGAATGGCAGAAG
TGATTAAACACGCTTTTATCTATGACAGAGCGTTTCTGGAGGAGCTGCTGAATATCCACTCACTGCGTGATATCACTAATGA
TCAGCTGAATGACATGATTTTTAAAGGCATTTCAATTAAAGCGTCTGTCGTTCAGCAGGATGAAAAAGAAGAGGGGATAAGA
GCTTACTTAAACTTTGGGCATACGCTCGGCCATGCCGTTGAGGCGGAATATGGATATGGGCAGATCACTCACGGTGATGCCG
TAGCCCTCGGAATGCAGTTTGCTTTATATATAAGCGAAAAGACTGTAGGCTGTGAAATGGACAGAAAACGTTTGGTCAGCTG
GCTAAAAAGCCTGGGTTATCCAAGTCAAATCAGAAAAGAGACGGAAACTTCGGTTCTCTTGAACCGTATGATGAATGATAAG
AAAACCCGTGGCGGGAAGATTCAGTTTATTGTGCTCAACGAATTAGGGAAAGTTGCTGATCATACATTTTCCAGAAATGAAC
TTGAGAGCTGGCTGAACAAATGGCGATTGGAGGAGACATCatgaTCACACTGGCTCACCTTCGGGTGGGCCTTTCTGCGTTT
ATATACTAGAGAGAGAATATAAAAAGCCAGATTATTAATCCGGCTTTTTTATTATTTAGGCAACTGAAACGATTCGGATCCT
GTATTACTATTCTTAgttaagatggcaagcttgacaagtatttccgacacatttacaatgaagttGGAGAAAAGATTAACTA
ATAAGGAGGACAAACATGAGAGTCCTGATCGTCGATAACTATGACAGCTTTACATTTAACCTGGCGACGTATGTCGAAGAAG
TTACAGGCCAAGCACCGACAGTTGTTAGAAACGATGATATTATCGATGAAACGCTGTTTGATGCGGTTATTCTGTCACCTGG
ACCGGGACATCCTGGCGTTCTGGCAGATTTTGGCATTTGCACAGGCATTATTGAAAGAGCACAAGTTCCGATTCTGGGCGTT
TGCCTGGGCCATCAAGGCATTGCACTGGCACATGGCGCAAGAGTTGAACTGGCACCGACACCGGTTCATGGCCAAGTTTCAA
CAATTTCACATAATGACAGCGCACTGTTTGACGCAATTCCGAGAGATTTTGATGTCGTCAGATATCATTCAATGATTGCATC
AGATCTGCCGGATTCAGTTGAAGCAACAGCATGGACAGCAGATGGCCTGATTATGGCACTGCAACATAAAACACTGCCGCAA
TGGGGAGTTCAATTTCATCCGGAATCAATTGGCGGACAATGGGGACATCAGATTATTCGCAATTTTCTTCATGCAGCACGCA
GCTATCATTGGGAAATTCAAGAAGAAGTTCTGGAAATTTCAGTCGATCCGGCAAGAGTTTTTGCAACACTGTATGGCGCAGC
AGAACAAGCATTTTGGCTGGATGATGCAGCAGGCACATCATATCTGGGAGATGCATCAGGACCGCTGGCAAGAACAAAAACA
TTTAGAGTTGGCGAAGGCGACTTTTTTGAATGGCTGGCAGCAGATCTGGCAAAAAATACAGTTGCACCTGGCGAAGGATTTA
GACTTGGCTGGGTTGGCTATGTTGGATATGAACTGAAAGCAGAATGCGGAGCACAAGCAGAACATAGAAGCAAACTGCCGGA
TGCACATCTGATTTTTGCAGATAGAGCACTGGCGATTGAAAAAGATAGAGTTAGACTGCTGTCACTGCAAGCAGATGCACAA
TGGTCAGCACAAGTCGAAGCAGCACTGAAACAACTGCAACCGGCACCGGCAGCACAAATCAAACCGATTGAACTGCAAGTTA
GAGATTCTCGCGAACAGTATCTGGATAAAATTGCAAAAGCGCAAGATCTGATTAGACGCGGAGAATCATATGAAATTTGCCT
GACAACACAACTGTCAGGCGAATGCACACAAGATCCGTTTGAACTGTATCTGGCACTGAGAGCAGAAAATCCGACAGCATAT
GGCTCATTTCTGAAATTTGGCGAAACAGCAATTCTGTCATCATCACCGGAACGCTTTATTACAATTGATGCAGGCGGAAGAG
TCGAATCAAAACCGATCAAAGGCACAAGAGGCAGAGGCAAAAATGCAGCAGAAGATGCAGAAATCATTAAAGAACTGCAGAG
CAATCCGAAAGATCGCGCTGAAAATCTGATGATTGTTGATCTGGTTAGAAATGATCTGGCGAGAGGCGCACAACCGATTACA
GTTAAAACAGAAAAACTGTTCGACGTGGAAACATTTGCAACAGTTCATCAACTGGTTTCAACAGTTAGCGCACAACTGGGCG

AAAAAAATGCAATTGGCTGCATTAGAGCAGCATTTCCAGGCGGATCAATGACAGGCGCTCCGAAACTGAGAACAATGGAAAT
TATTGATGCACTGGAAGCGGCACCGAGAGGCATTTATTCAGGCGGACTGGGCTATTTTTCACTGGATGGCTCAGTTGATCTG
AGCATGGTTATTAGAACACTGGTTCTGCATGCTGGCCATCTGGAATATGGCGTTGGCGGAGCAATTCTTGCACTGTCAGATC
CGGCTGAAGAATGGGAAGAAATTCGCATTAAATCAACACCGCTGCTGAAACTGTTTGGCGTCGAATTTCCTGGAGGATCAGG
CGGAGGCGGAAGCGGAGGC**TGCTGTCCGGGATGCTGC**GGAGGATCTGGTGGCGGAGGCTCTGGCGGAATGTATTGGATTAAT
GGCAAACCGTGCAATCAACTGCCGGTTAATGATAGAGCAGTCCAATTTGGAGATGGCTGCTTTACAACAATTAGAGTCGAAC
AAGGCCAAGCGGCACTGCTGCCGCTGCATATTAAACGCCTGCAAAAAGGCGTTGAAAAGTTATTTATGCCTGCACTGGATTG
GCTTCAGCTGGAAGATCATATCAAACAAGTTGCAACAGGCTGCGAATCAGGCGTTCTGAAAGTTATTCTTTCAAGAGGCGTT
GGAGGCCGTGGCTATGGCATTTCAGATGCAATTGAACCGAATCAAGTTCTGTCATTAAGCAGCTATCCGGAACAATATGTCA
TTCAACGCAAAAATGGCATTAGCCTGGTTCTTTCACCGATTGTTATGGGCATTAATCCGCATCTGGCAGGCATTAAACATCT
GAATAGACTGGAACAGGTTCTGATCAAACGGTTTATCGAACAAAGCAAAGCAGATGAAGCACTGGTCCTGGATTCAGATGGA
CTGCTGGTTGAATGCTGCACAGCAAACATTTTTTGGCGCAAAGGCAAAAACGTTTATACACCGGATCTTAATCAATGCGGAG
TTGAAGGCGTTATGCGCCAGAAAATTATGCAACTGCTGGCAGAAAGCGATTATAACCTGTCATGCGTTATGAGATATCCGGA
AGTTCTTGCACATGCGGATGAAGTCATTATTTGCAATTCACTGATGCCGGTCATTGCGGTCAATCAAATTCAAGCGCATAAA
AATCAACCGGCATGGAAATATCAATCACGCGAACTGCATGAATATCTGCTTCCGGCATGCCTGAGACTGTAATAATAAAAGT
CTCGTAAAGCGTTCTATCAATAACCCGTTGGTGCCAGGCATCAAATAAAACGAAAGGCTCAGTCGAAAGACTGGGCCTTTCG
TTTTATCTGTTGTTTGTCGGTGAACGCTCTCTACTAGAGTCACACTGGCTCACCTTCGGGTGGGCCTTTCTGCGTTTATAgc
aggcgagaaaggagagagaacgcaaggagaggcacgcgagggaggaaaggcaggataccgttcgtatagcatacattatacg
aagttatgaattccgaaaaaacggttgcatttaaatcttacatatgtaatactttcaaagactactagtacataaggaggaa
ctact**ATGAACGAGAAAAATATAAAACACAGTCAAAACTTTATTACTTCAAAACATAATATAGATAAAATAATGACAAATAT**
AAGATTAAATGAACATGATAATATCTTTGAAATCGGCTCAGGAAAAGGCCATTTTACCCTTGAATTAGTAAAGAGGTGTAAT
TTCGTAACTGCCATTGAAATAGACCATAAATTATGCAAAACTACAGAAAATAAACTTGTTGATCACGATAATTTCCAAGTTT
TAAACAAGGATATATTGCAGTTTAAATTTCCTAAAAACCAATCCTATAAAATATATGGTAATATACCTTATAACATAAGTAC
GGATATAATACGCAAAATTGTTTTTGATAGTATAGCTAATGAGATTTATTTAATCGTGGAATACGGGTTTGCTAAAAGATTA
TTAAATACAAAACGCTCATTGGCATTACTTTTAATGGCAGAAGTTGATATTTCTATATTAAGTATGGTTCCAAGAGAATATT
TTCATCCTAAACCTAAAGTGAATAGCTCACTTATCAGATTAAGTAGAAAAAAATCAAGAATATCACACAAAGATAAACAAAA
GTATAATTATTTCGTTATGAAATGGGTTAACAAAGAATACAAGAAAATATTTACAAAAAATCAATTTAACAATTCCTTAAAA
CATGCAGGAATTGACGATTTAAACAATATTAGCTTTGAACAATTCTTATCTCTTTTCAATAGCTATAAATTATTTAATAAGT
AATAGggggatcttctcgagataacttcgtatagcatacattatacgaacggtagagagagcacagatacggcgacgacacc
gaagcagagcgaagcagtgacaggagcctcgTACGATAAGAACAGCTTAGAAATACACAAGAGTGTGTATAAAGCAATTAGA
ATGAGTTGAGTTAGAGAATAGGGTAGCAGAGAATGAGTTTAGTTGAGCTGAGACATTATGTTTATTCTACCCAAAAGAAGTC
TTTCTTTTGGGTTTATTTGTTATATAGTATTTTATCCTCTCATGCCATCTTCTCATTCTCCTTGCCATAAGGAGTGAGAGCA
atgAATTTCCAATCAAACATTTCCGCATTTTTAGAGGACAGCTTGTCCCACCACACGATACCGATTGTGGAGACCTTCACAG
TCGATACACTGACACCCATTCAAATGATAGAGAAGCTTGACAGGGAGATTACGTATCTTCTTGAAAGCAAGGACGATACATC
CACTTGGTCCAGATATTCGTTTATCGGCCTGAATCCATTTCTCACAATTAAAGAAGAGCAGGGCCGTTTTTCGGCCGCTGAT
CAGGACAGCAAATCTCTTTACACAGGAAATGAACTAAAAGAAGTGCTGAACTGGATGAATACCACATACAAAATCAAAACAC
CTGAGCTTGGCATTCCTTTTGTCGGCGGAGCTGTCGGGTACTTAAGCTATGATATGATCCCGCTGATTGAGCCTTCTGTTCC
TTCGCATACCAAAGAAACAGACATGGAAAAGTGTATGCTGTTTGTTTGCCGGACATTAATTGCGTATGATCATGA

PCR mix:

A1 / A2	2 × 2.5 µL primer (10 µM, ≙ 0.5 µM final), 0.2 µL template (≈ 10 ng pUC19-A), 1 µL DMSO, 25 µL Q5 master-mix, 18 µL H_2O, (50 µL final)
B1 (F2-F1-pUC19-F5-F3+4)	2 × 10 µL primer (10 µM, ≙ 1 µM final), 1 µL template (≈ 100 ng pUC19-B), 4 µL DMSO, 50 µL Q5 master-mix, 25 µL H_2O, (100 µL final)

PCR conditions:

A1 F5-BB-F1-F2	20 sec @ 98°C – 32× (10 sec @ 98°C, 20 sec @ **52**°C-**0.1**°C /cycle, 6 min @ 72°C) – 12 min @ 72°C – hold @ 4°C
A2 F3+4	20 sec @ 98°C – 32× (10 sec @ 98°C, 20 sec @ **53**°C-**0.1**°C /cycle, 1 min @ 72°C) – 4 min @ 72°C – hold @ 4°C
B1 F2-F1-pUC19-F5-F3+4	30 sec @ 98°C – 32× (10 sec @ 98°C, 20 sec @ **72**°C-**0.4**°C /cycle, 6 min @ 72°C) – 12 min @ 72°C – hold @ 4°C

Assembly mix:

Vector A^{tagged}	Vector B^{tagged}
A1 + A2 + gBlock5 + gBlock6	B1 + gBlock4
0.14 pmol each fragment	0.12 pmol vector, 0.24 pmol insert

10 µL DNA + 10 µL NEBuilder ➔ 20 µL total volume

Sequencing & Colony PCR (gene & linker sequence spanning**):**

A^{tagged}

frag2for (pabAB) – frag2rev → 2200 bp (T_a = 60°C)
verC2 – verB3 (ermHI) → 1500 bp (T_a = 52°C)

B^{tagged}

frag2for – verB2 → 2300 bp (T_a = 53°C)
verC2 – verB3 (ermHI) → 1400 bp (T_a = 52°C)

<u>Integration and verification of synthetic operons</u>

Primers:

frag 1 for (aroB): GACGCTCTTGGCGCATGACAGC (T_m = 62°C)
frag 5 rev (aroH): TCATGATCATACGCAATTAATGTCCGGC (T_m = 60°C)

verB (rev, pabAB): CCAGATATGATGTGCCTGCTGC (T_m = 57.4°C)
verC (for, pabC): GGTCAATCAAATTCAAGCGC (T_m = 51°C)

verA (for, aroB): TGCAGAGTGAAGCGATTCGT (T_m = 55.6°C)
verD (rev, trpE): CTCTCCAGTGAGCCTTGC (T_m = 52.4°C)

PCR mix:

<u>Vector A/B</u>	2 × 5 µL primer (10 µM, ≙ 0.5 µM final), 1 µL template (≈ 100 ng pUC19-A/B), 4 µL DMSO, 50 µL Q5 master-mix, 36 µL H_2O, (100 µL final)
<u>“out-in”</u> / <u>“in-out”</u>	2 × 5 µL primer (10 µM, ≙ 0.5 µM final), 1 µL template (≈ 35 ng gDNA), 4 µL DMSO, 50 µL Q5 master-mix, 36 µL H_2O, (100 µL final)
<u>“locus spanning”</u>	2 × 10 µL primer (10 µM, ≙ 1 µM final), 1 µL template (≈ 35 ng gDNA), 4 µL DMSO, 50 µL Q5 master-mix, 26 µL H_2O, (100 µL final)

PCR conditions:

<u>Vector A/B</u> frag 1 for & frag 5 rev	20 sec @ 98°C – 32× (10 sec @ 98°C, 20 sec @ **70**°C-**0.3**°C /cycle, 4 min @ 72°C) – 8 min @ 72°C – hold @ 4°C
<u>“out-in”</u> verA-verB	30 sec @ 98°C – 48× (10 sec @ 98°C, 20 sec @ **58**°C-**0.2**°C /cycle, 2 min @ 72°C) – 4 min @ 72°C – hold @ 4°C
<u>“in-out”</u> verC-verD	30 sec @ 98°C – 48× (10 sec @ 98°C, 20 sec @ **53**°C-**0.2**°C /cycle, 2 min @ 72°C) – 4 min @ 72°C – hold @ 4°C
<u>“locus spanning”</u> verA-verD	30 sec @ 98°C – 48× (10 sec @ 98°C, 20 sec @ **56**°C-**0.2**°C /cycle, 4 min @ 72°C) – 8 min @ 72°C – hold @ 4°C
