# Supplementary material for: Metabolic engineering of Bacillus subtilis for production of para‐aminobenzoic acid – unexpected importance of carbon source is an advantage for space application
Source: Microb Biotechnol. 2019 Apr 13;12(4):703–14. doi: 10.1111/1751-7915.13403 (PMC6559200; doi:10.1111/1751-7915.13403)
Supplement: Supplementary file 2 — Appendix S2. Cultivation data containing growth‐curves and development of the pH. [file MBT2-12-703-s002.pdf]

Growth, pH & production data

In the following (pages 1-4) the growth of certain mutant *B. subtilis* strains on media containing different (concentrations of) carbon- and / or nitrogen-sources is visualised by plotting the time against optical density. Where applicable the development of the pH throughout the cultivation has been included as well.

Further (page 5), the growth data of certain mutant *B. subtilis* strains is correlated with the pABA produced over time throughout two representative cultivations.

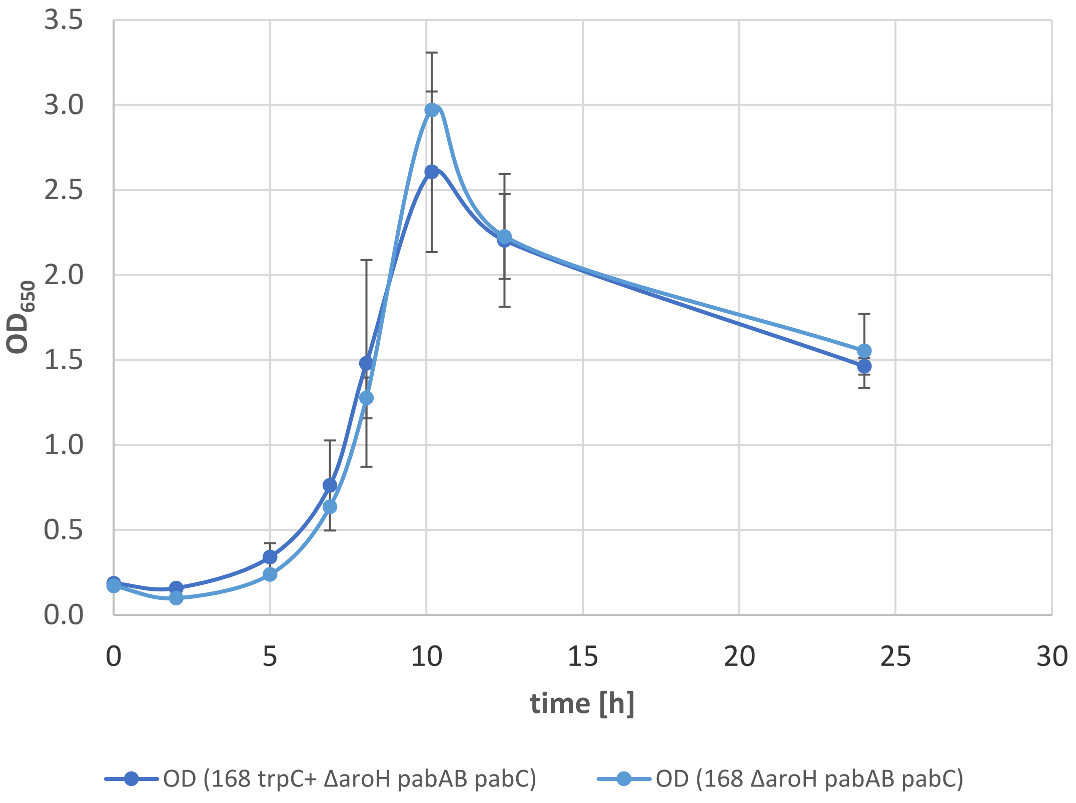

168 Δ*aroH pabAB pabC* & 168 *trpC*+ Δ*aroH pabAB pabC*, 0.1665 C-mol/L glucose, 18.7 mM total N

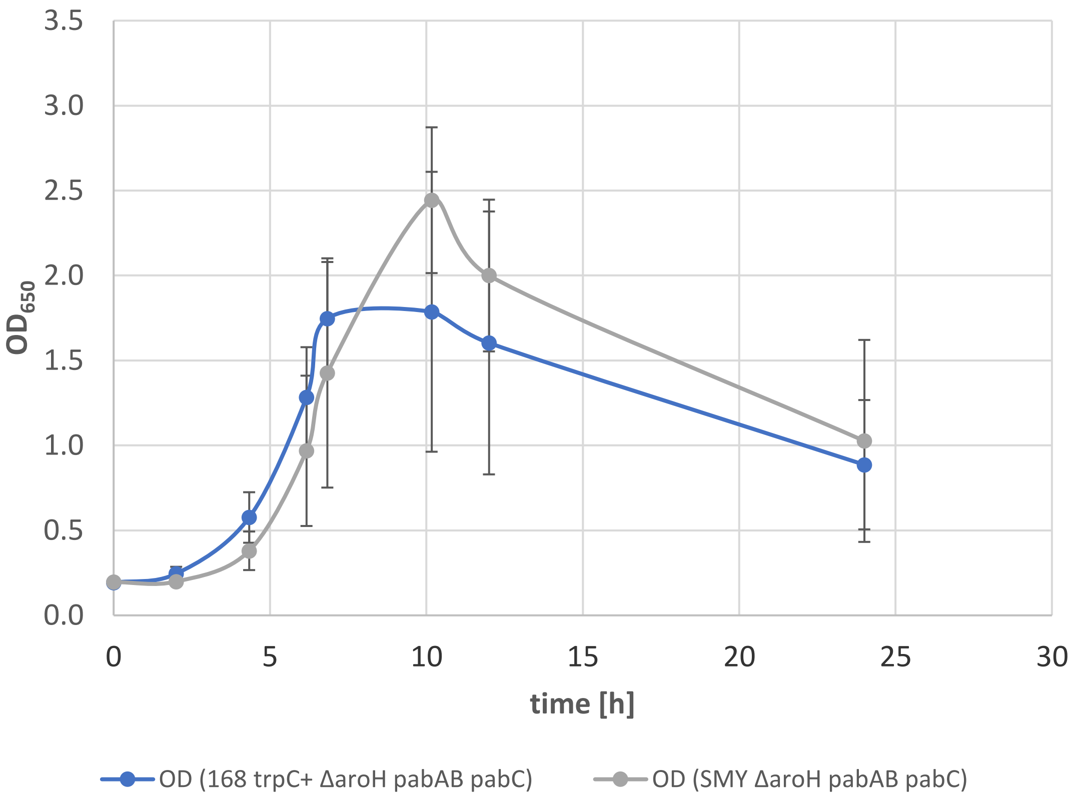

168 *trpC*+ Δ*aroH pabAB pabC* & SMY Δ*aroH pabAB pabC*, 0.1665 C-mol/L glucosamine, 43.8 mM total N

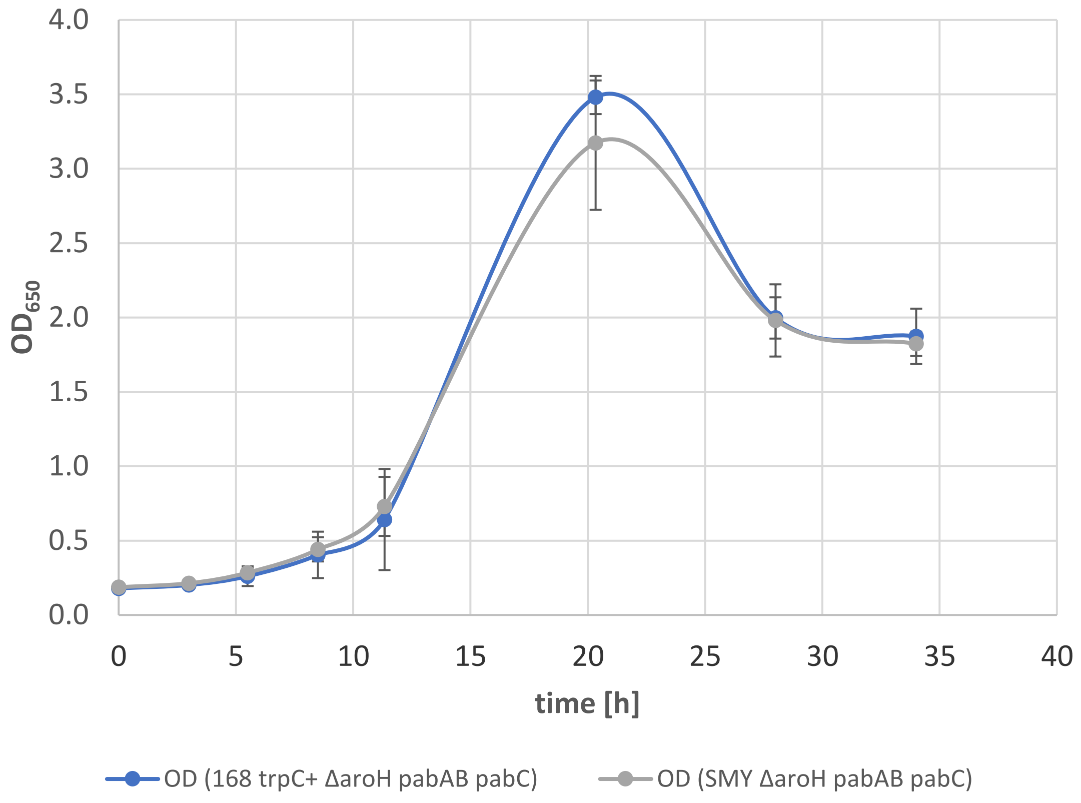

168 *trpC*⁺ Δ*aroH pabAB pabC* & SMY Δ*aroH pabAB pabC*, 0.1665 C-mol/L acetylglucosamine, 39.5 mM total N

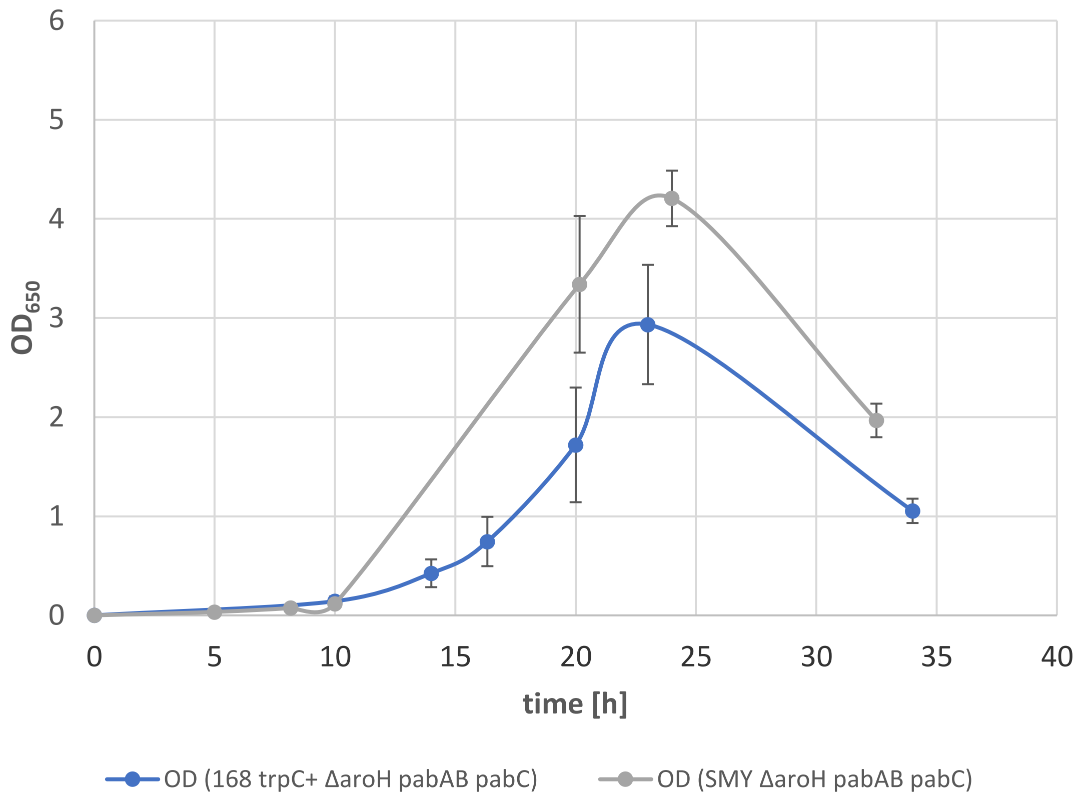

168 *trpC*⁺ Δ*aroH pabAB pabC* xyl⁺ & SMY Δ*aroH pabAB pabC* xyl⁺, 0.1665 C-mol/L xylose, 18.7 mM total N

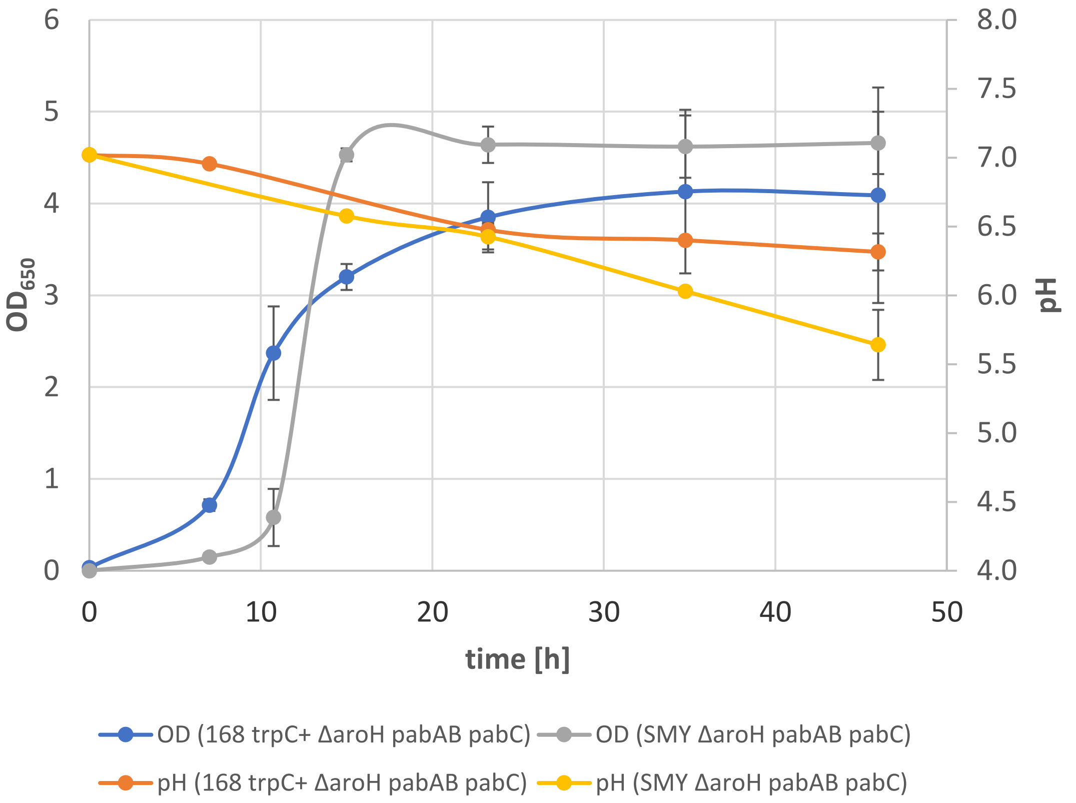

168 *trpC*⁺ Δ*aroH pabAB pabC* & SMY Δ*aroH pabAB pabC*, 0.667 C-mol/L glycerol, 18.7 mM total N

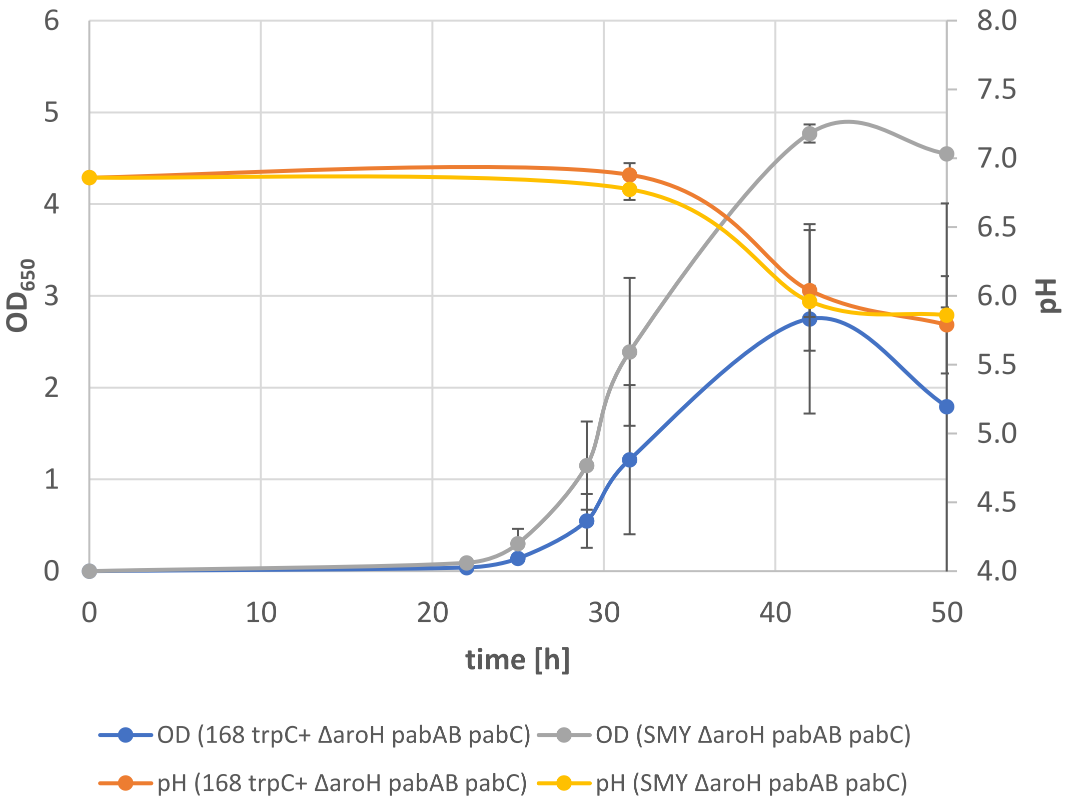

168 *trpC*⁺ Δ*aroH pabAB pabC* xyl⁺ & SMY Δ*aroH pabAB pabC* xyl⁺,0.667 C-mol/L xylose, 18.7 mM total N

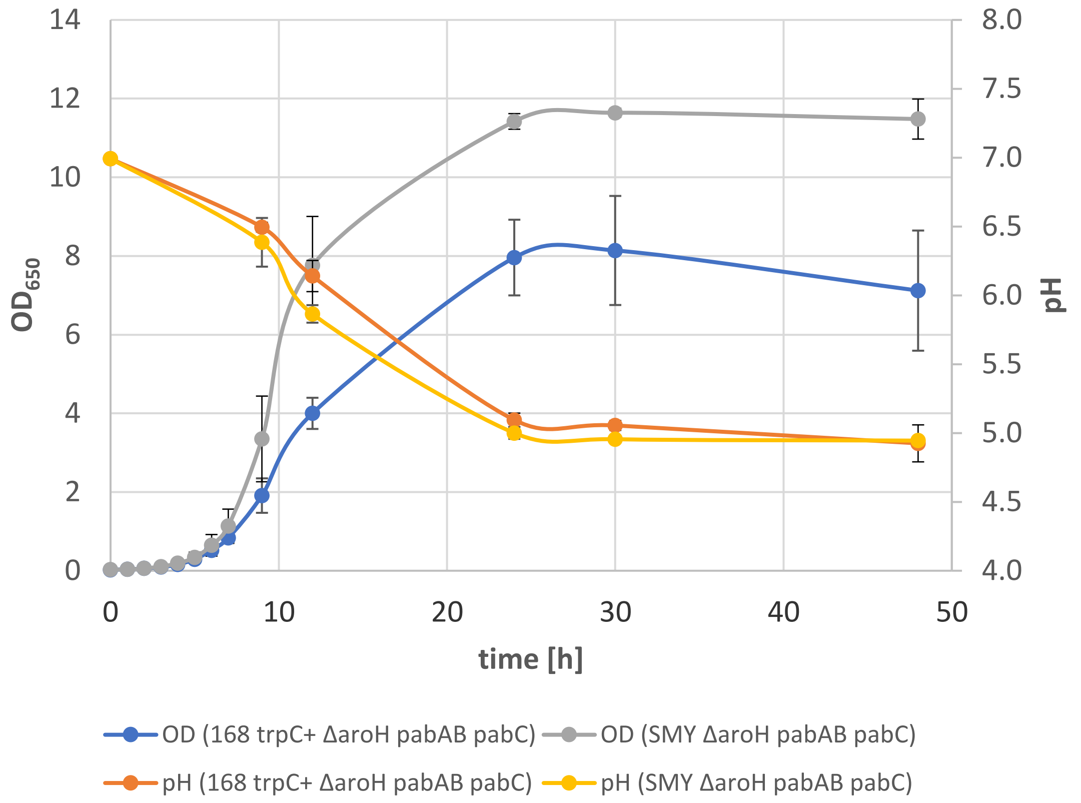

168 *trpC*[+] Δ*aroH pabAB pabC* & SMY Δ*aroH pabAB pabC*, 0.667 C-mol/L sucrose, 100 mM total N

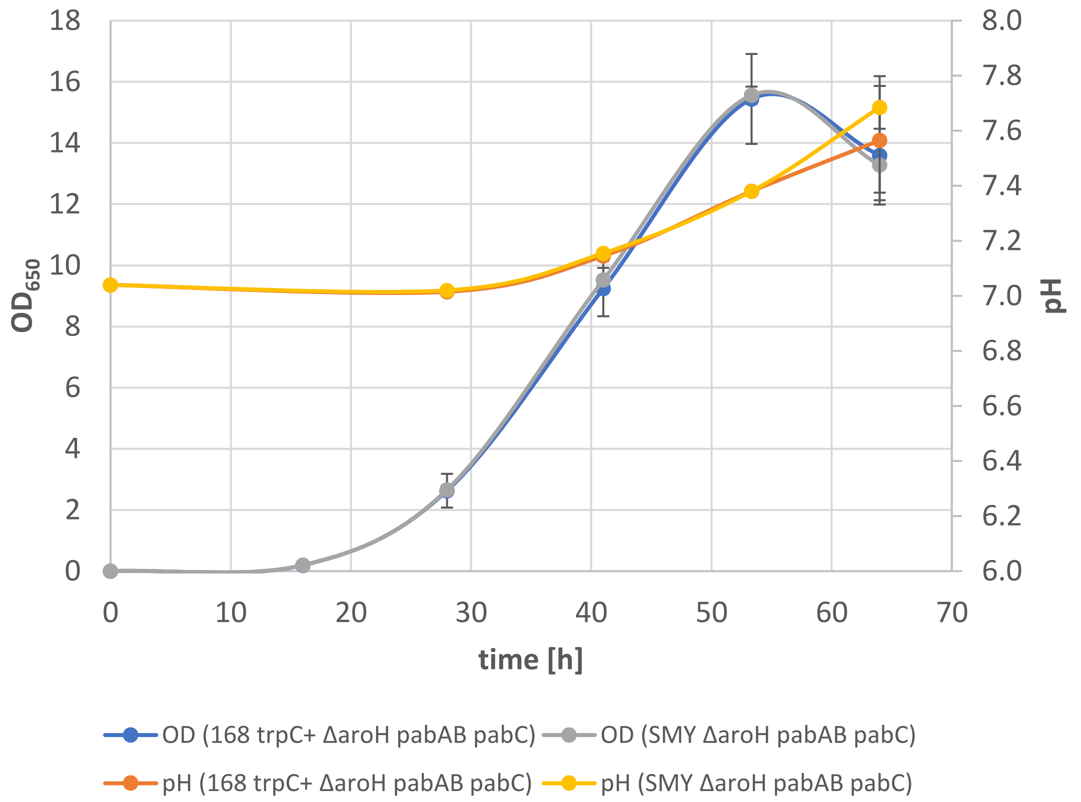

168 *trpC*[+] Δ*aroH pabAB pabC* & SMY Δ*aroH pabAB pabC*, 0.667 C-mol acetylglucosamine, 101.9 mM total N

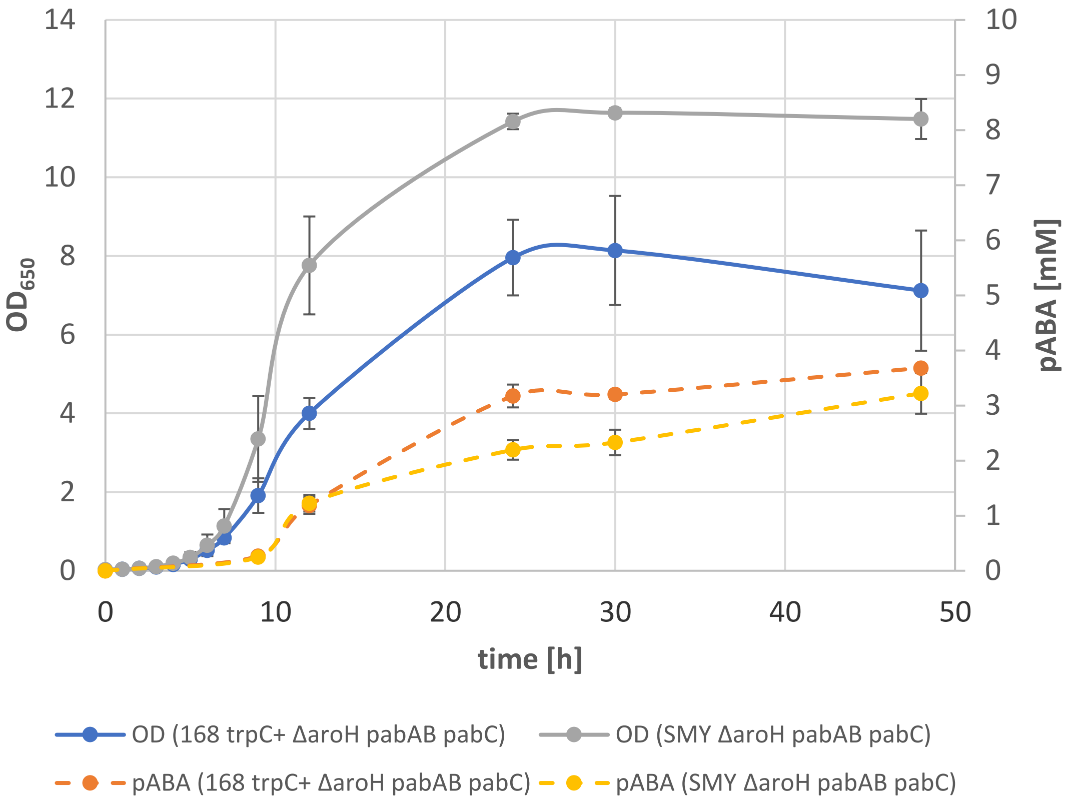

168 *trpC*[+] *ΔaroH pabAB pabC* & SMY *ΔaroH pabAB pabC*, 0.667 C-mol/L sucrose, 100 mM total N

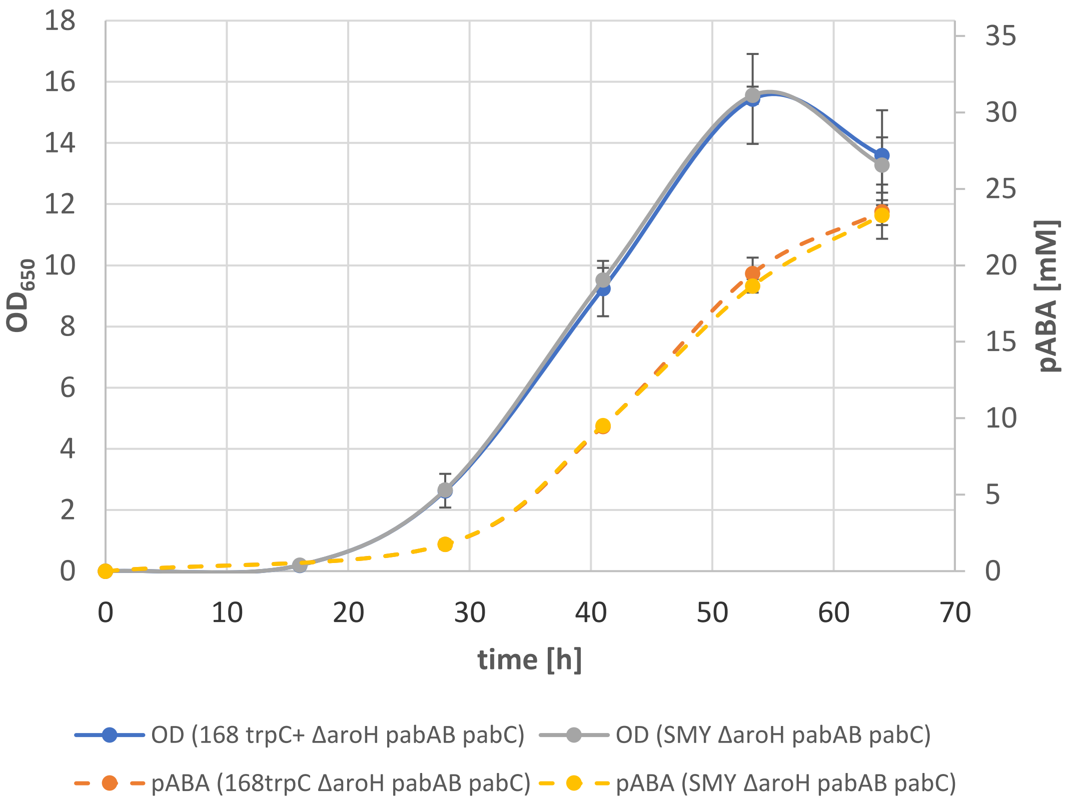

168 *trpC*[+] *ΔaroH pabAB pabC* & SMY *ΔaroH pabAB pabC*, 0.667 C-mol acetylglucosamine, 101.9 mM total N
