# Supplementary material for: Metabolic engineering of Bacillus subtilis for production of para‐aminobenzoic acid – unexpected importance of carbon source is an advantage for space application
Source: Microb Biotechnol. 2019 Apr 13;12(4):703–14. doi: 10.1111/1751-7915.13403 (PMC6559200; doi:10.1111/1751-7915.13403)
Supplement: Supplementary file 3 — Appendix S3. Protocol of aminobenzoate‐assay and example standard‐curves. [file MBT2-12-703-s003.pdf]

Protocol for quantification of aminobenzoates

1) Prepare pABA standards[1] (serial dilution): 64 µM, 32 µM, 16 µM, 8 µM, 4 µM, 2 µM, 1 µM
2) Add 200 µL of 5 M NaOH to 1 mL of pABA solution / sample, boil for 1 – 2 h (not-essential).
3) Add 300 µL (100 µL if the previous step is skipped) of 5 M HCl to bring pH to < 2
4) Add 100 µL of 14.5 mM $NaNO_2$ (sodium nitrite) solution, wait 2 – 20 min
5) Add 100 µL of 44 mM $H_6N_2O_3S$ (ammonium sulfamate) solution, wait 2 – 3 min
6) Add 100 µL of 4 mM N-(1-naphthyl)-ethylenediamine dihydrochloride solution[2], wait 20 min
7) Measure absorbance at 540 nm (linear up to ≈ 40 µM – if significantly higher, dilute sample)

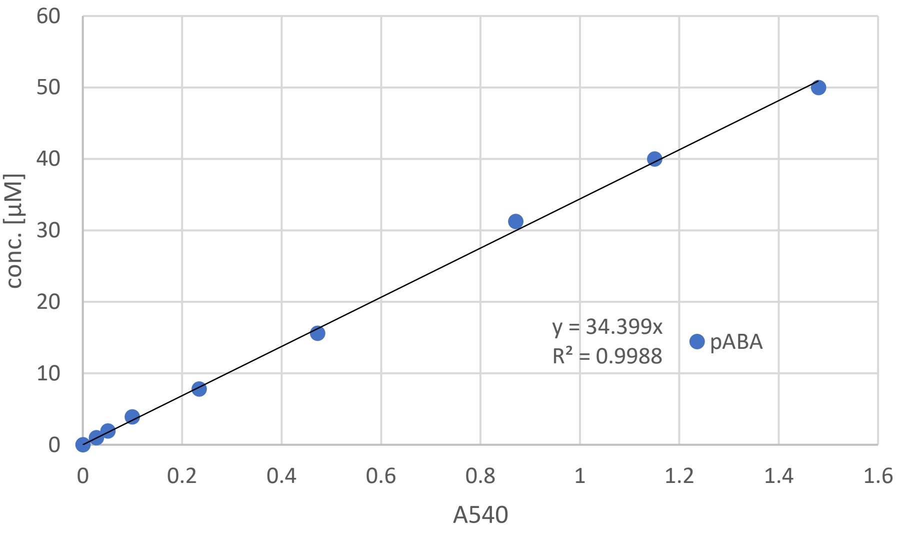

Correlation of pABA concentration and absorption at 540 nm in H_2O

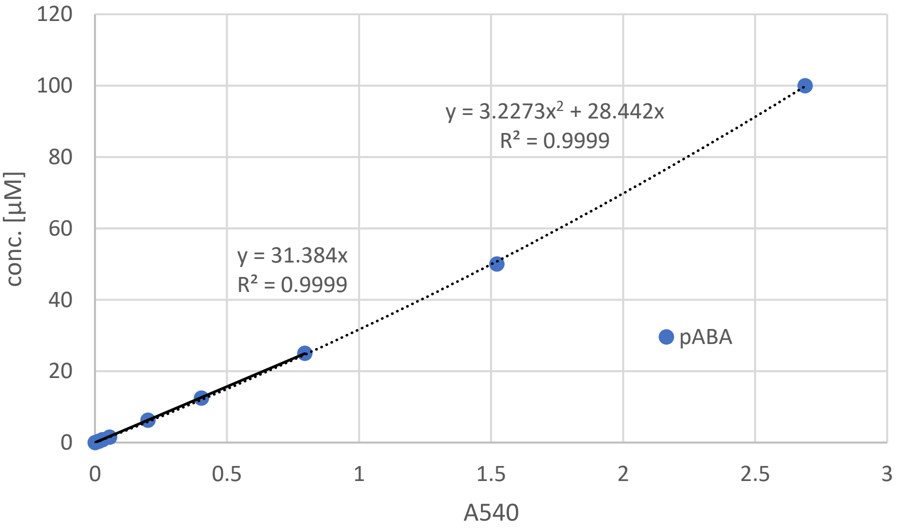

Correlation of pABA concentration and absorption at 540 nm in M9 medium

---

[1] Pigment will precipitate eventually above pABA concentrations of ≈ 100 µM (after 24 h or earlier). mABA, oABA, benzocaine and 4-amino-phenylalanine form stable azo-dyes at concentrations of 1 mM, potentially also higher.
[2] Solution slowly degrades at room temperature, which impacts dye stability and affects the calibration curve.

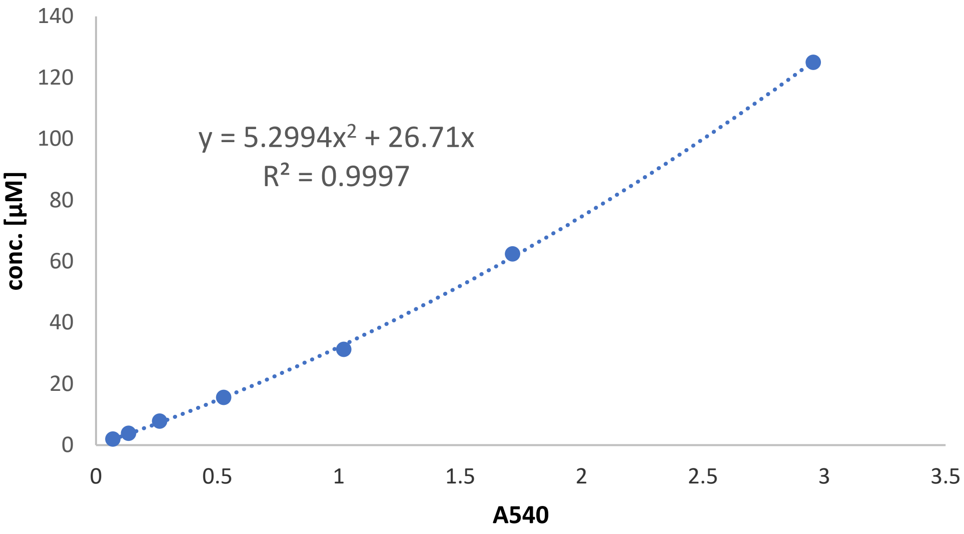

Correlation of mABA concentration and absorption at 540 nm in H_2O

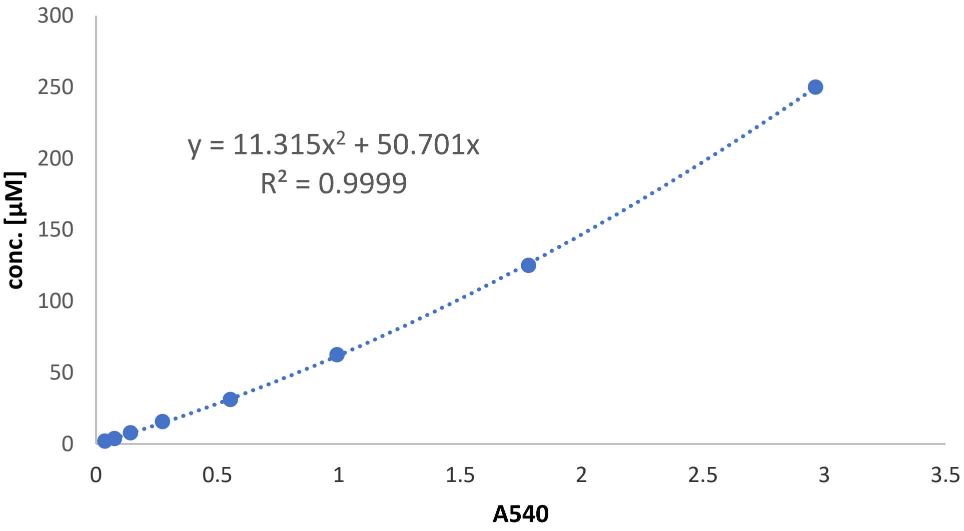

Correlation of oABA concentration[3] and absorption at 540 nm in H_2O

[3] Formation of pigment much slower, full colour development only after >20 min.

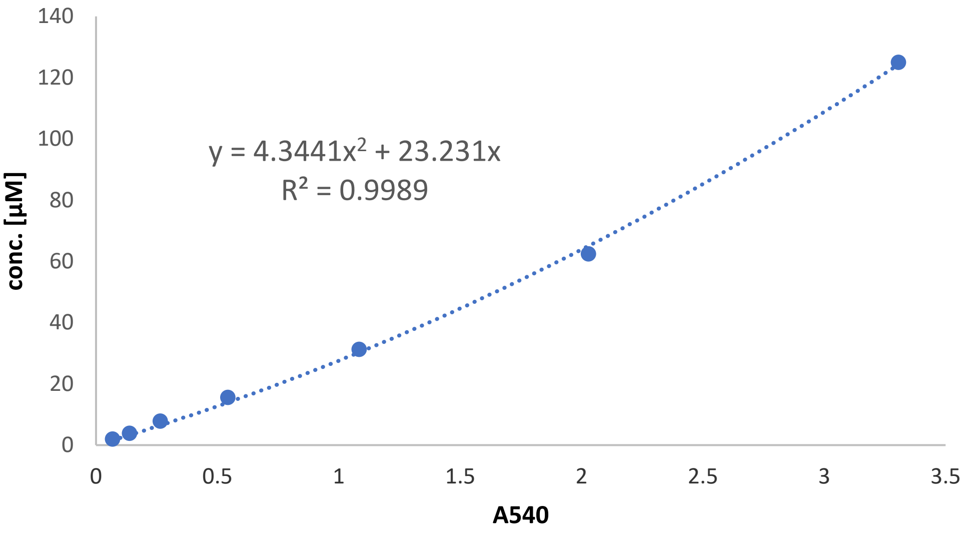

Correlation of benzocaine concentration and absorption at 540 nm in H_2O

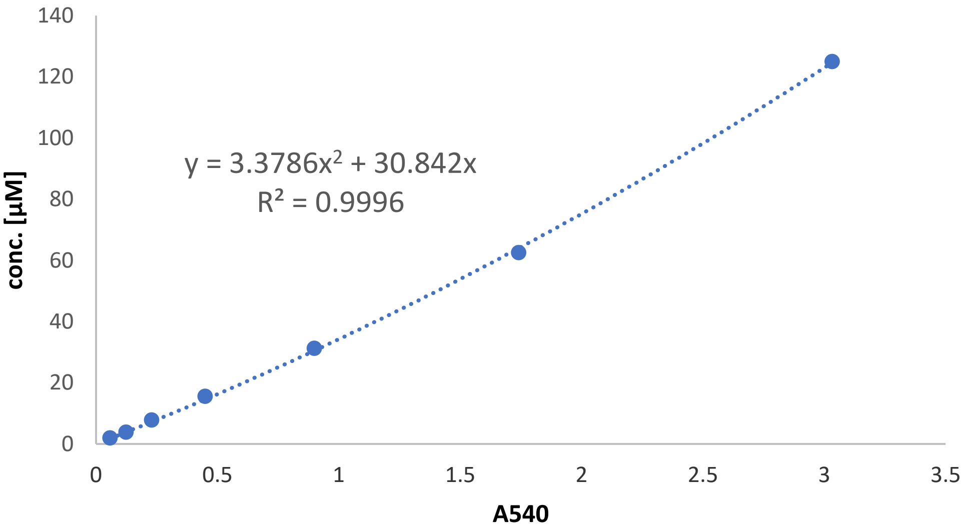

Correlation of 4-amino-phenylalanine[4] concentration and absorption at 540 nm in H_2O

[4] Formation of pigment much slower, full colour development only after >60 min.

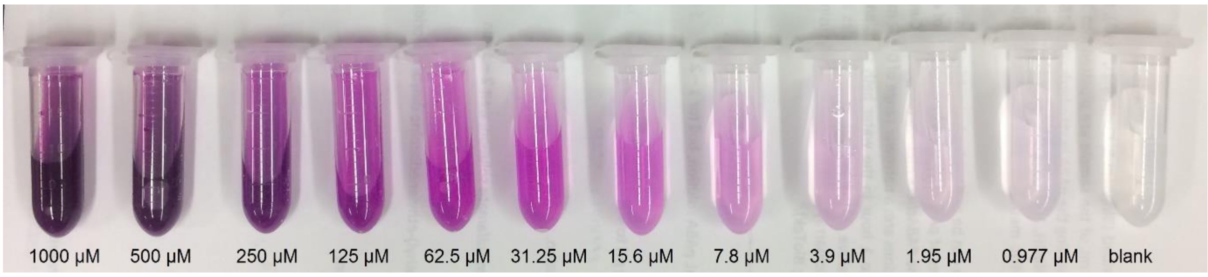

aminobenzoate assay 0h (pABA)

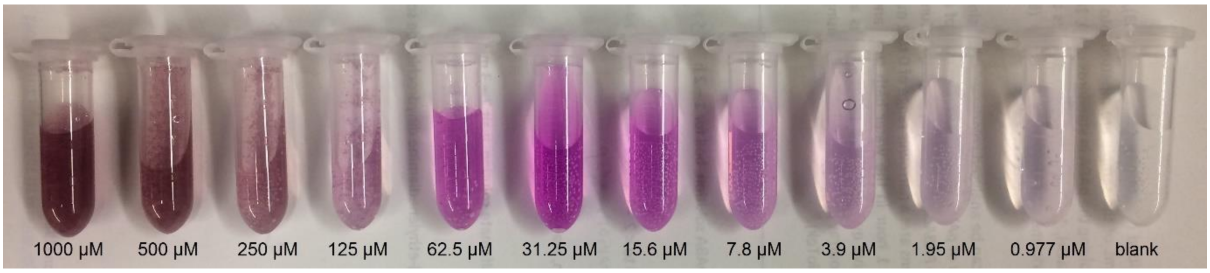

aminobenzoate assay 16h (pABA)

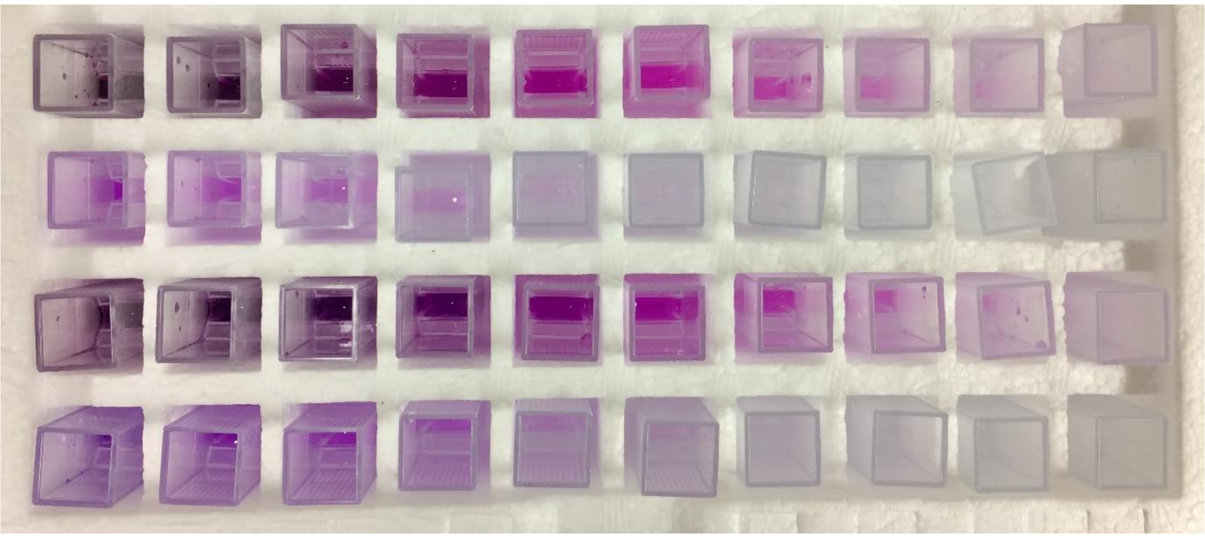

aminobenzoate assay 10 min (mABA, oABA, benzocaine, 4-amino-phenylalanine)

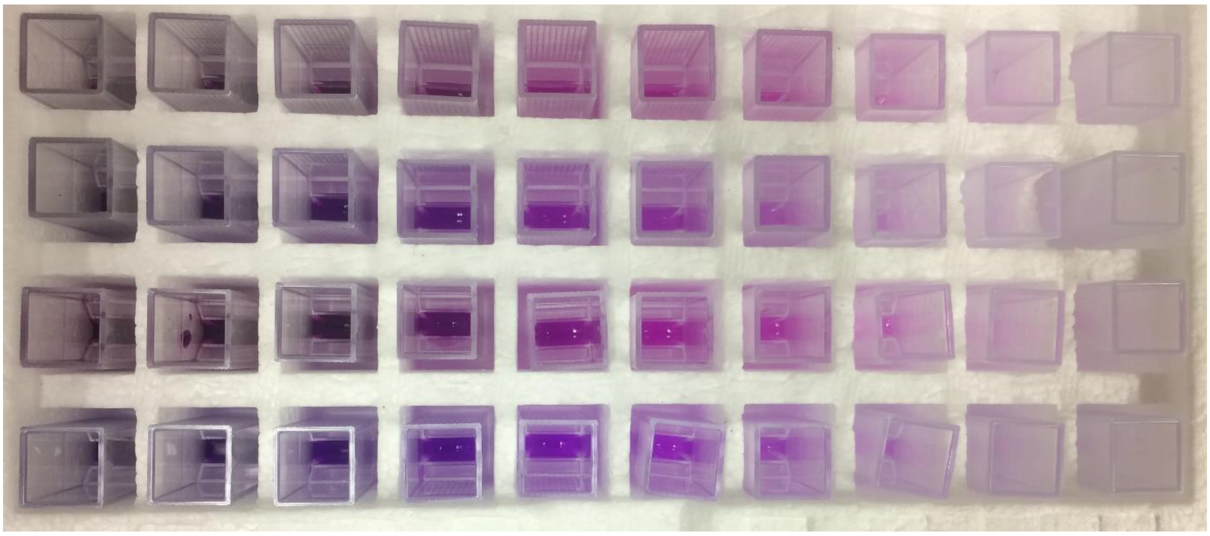

aminobenzoate assay 90 min (mABA, oABA, benzocaine, 4-amino-phenylalanine)
