# Supplementary material for: Metabolic engineering of Bacillus subtilis for production of para‐aminobenzoic acid – unexpected importance of carbon source is an advantage for space application
Source: Microb Biotechnol. 2019 Apr 13;12(4):703–14. doi: 10.1111/1751-7915.13403 (PMC6559200; doi:10.1111/1751-7915.13403)

Detection of pab-proteins from different phases of cultivation on protein gels

Fluorescence staining (Lumio™) of tagged enzymes for formation of pABA from chorismate in *B. subtilis* 168 from crude-protein extract for quantification. M_w of pab-proteins (calculated): $pabAB^{tagged}$ 68.5 kDa, $pabC^{tagged}$ 31.74 kDa, $pabABC^{tagged}$ 100.11 kDa.

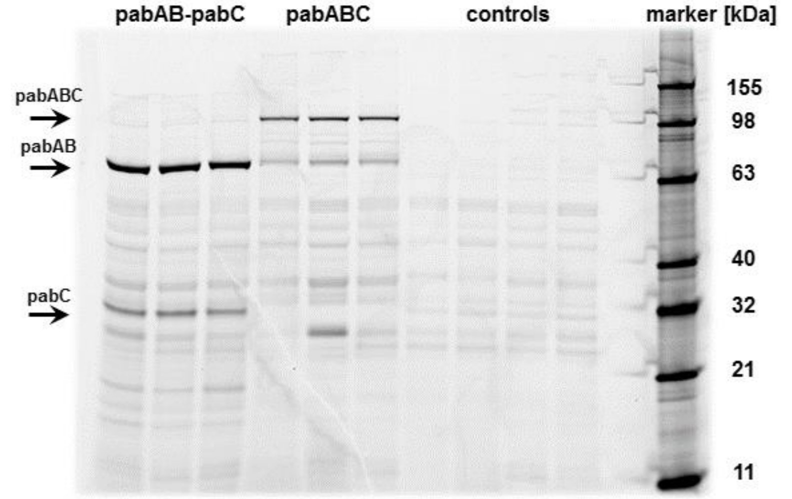

exponential-phase (9h) samples from growth on sucrose

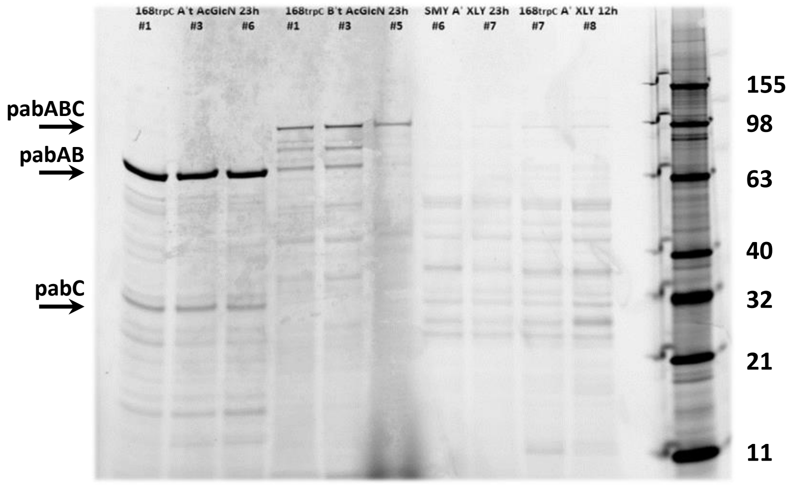

exponential-phase (23h) samples from growth on acetyl-glucosamine

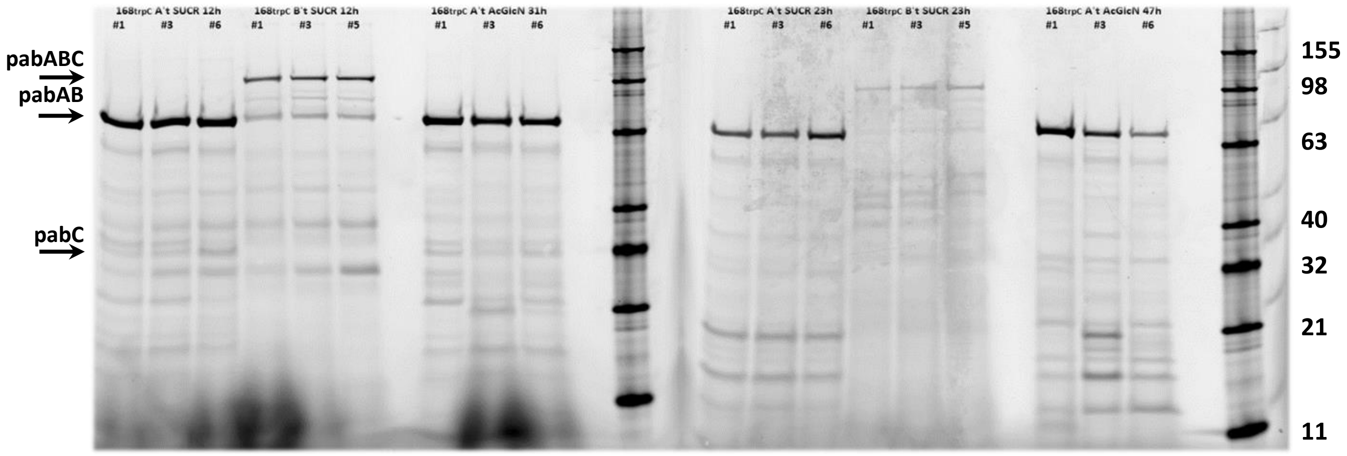

late exponential-phase (12h) & stationary-phase (23h) samples from growth on sucrose

Expression levels of aminodeoxychorismate lyase and aminodeoxychorismate synthase/lyase fusion-protein enzyme on different carbon-sources / stages of cultivation, relative to aminodeoxychorismate synthase.

carbon-source & growth-phase	*pabC* relative to *pabAB* [fold]	*pabABC* relative to *pabAB* [fold]	*pabABC* + *pabAB* fragment relative to *pabAB* [fold]
SUCR 9h (mid. exp.-phase)	0.37±0.06	0.49±0.05	0.68±0.09
SUCR 12h (late exp.-phase)	0.15±0.04	0.50±0.08	0.71±0.13
SUCR 23h (stat.-phase)	0.38±0.07	0.30±0.01	0.45±0.05
AcGlcN 23h (mid. exp.-phase)	0.28±0.02	0.34±0.08	0.54±0.16
average SUCR	0.30±0.11	0.43±0.10	0.61±0.14
average exp.-phase	0.32±0.06	0.41±0.10	0.61±0.14
overall average	0.29±0.09	0.41±0.10	0.60±0.15

Relative activities of aminodeoxychorismate synthase/lyase fusion-protein enzyme on different carbon-sources, calculated as the quotient of fold pABA production and fold enzyme expression.

carbon-source	*pabABC* [fold]	*pabABC* + *pabAB* fragment [fold]
SUCR	1.15	0.81
AcGlcN	1.12	0.70
overall	1.10±0.02	0.75±0.07
average	0.92±0.25	

Adaptive evolution of *Bacillus* strains to growth on xylose and selection of mutants

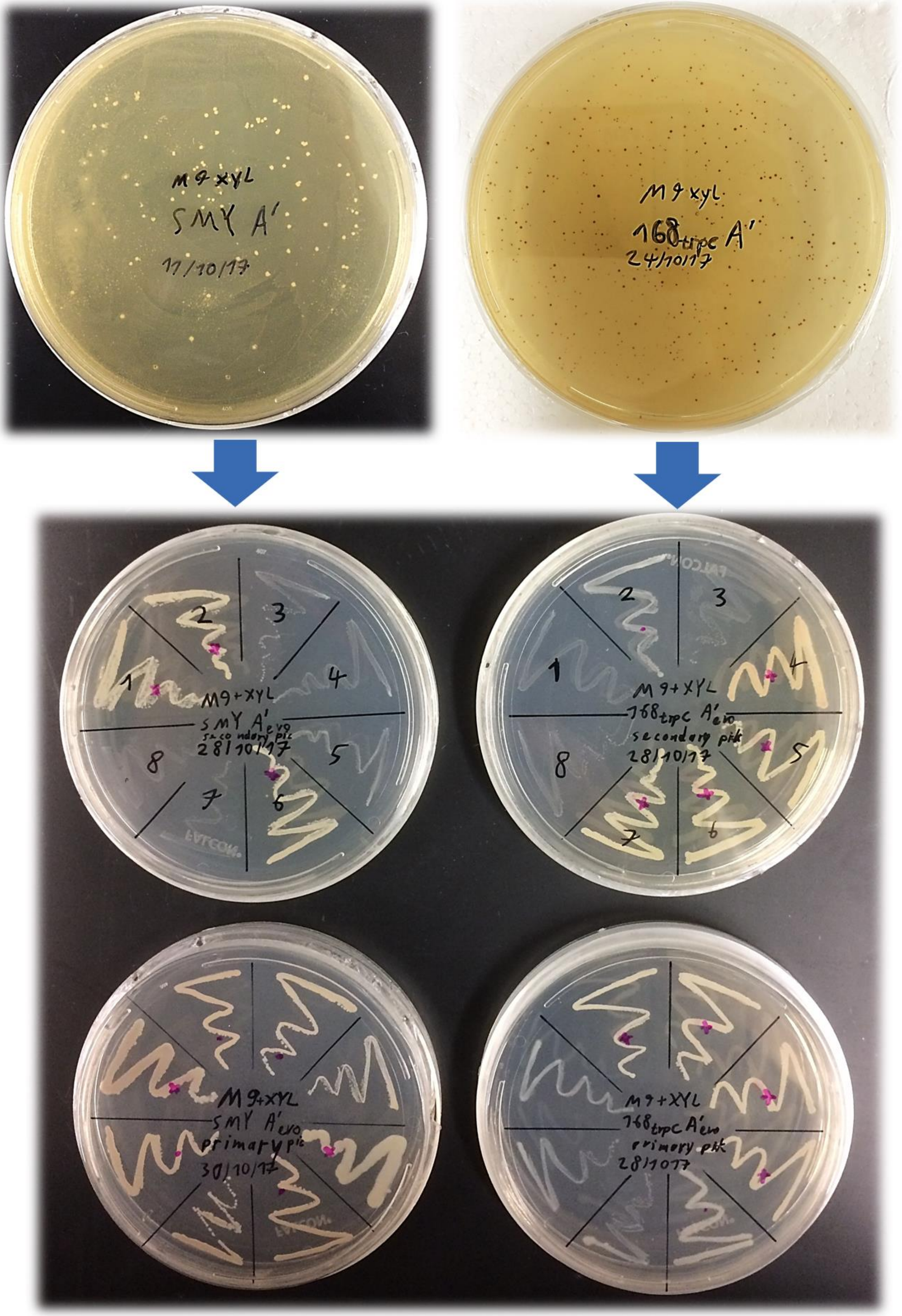

Supplement: Supplementary file 4 — Appendix S4. Additional data of protein quantification and figures of xyl+ mutants. [file MBT2-12-703-s004.pdf]
